# Antecedents predicting digital contact tracing acceptance: a systematic review and meta-analysis

**DOI:** 10.1186/s12911-023-02313-1

**Published:** 2023-10-11

**Authors:** Kuang-Ming Kuo

**Affiliations:** Department of Business Management, National United University, No.1, 360301 Lienda, Miaoli Taiwan, Republic of China

**Keywords:** COVID-19, Digital contact tracing, Subgroup analysis, Systematic review and meta-analysis, Antecedents, Moderators

## Abstract

An awareness of antecedents of acceptance of digital contact tracing (DCT) can enable healthcare authorities to design appropriate strategies for fighting COVID-19 or other infectious diseases that may emerge in the future. However, mixed results about these antecedents are frequently reported. Most prior DCT acceptance review studies lack statistical synthesis of their results. This study aims to undertake a systematic review and meta-analysis of antecedents of DCT acceptance and investigate potential moderators of these antecedents. By searching multiple databases and filtering studies by using both inclusion and exclusion criteria, 76 and 25 studies were included for systematic review and meta-analysis, respectively. Random-effects models were chosen to estimate meta-analysis results since *Q*, *I* ^*2*^, and *H* index signified some degree of heterogeneity. Fail-safe N was used to assess publication bias. Most DCT acceptance studies have focused on DCT related factors. Included antecedents are all significant predictors of DCT acceptance except for privacy concerns and fear of COVID-19. Subgroup analysis showed that individualism/collectivism moderate the relationships between norms/privacy concerns and intention to use DCT. Based on the results, the mean effect size of antecedents of DCT acceptance and the potential moderators may be more clearly identified. Appropriate strategies for boosting the DCT acceptance rate can be proposed accordingly.

**Supplementary Information:**

The online version contains supplementary material available at 10.1186/s12911-023-02313-1.

## Introduction

Infectious diseases such as SARS, Ebola, and COVID-19 demand rapid response and targeted control measures [1]. COVID-19, induced by severe acute respiratory syndrome Coronavirus 2, has posed an immense global challenge, and it has been declared to be pandemic after March, 2020 [2]. Though pharmaceutical control methods such as vaccines are expected to show efficacy, they are often not readily available within a short period of time [3]. Non-pharmaceutical control measures such as contact tracing, social distancing, or testing and isolating infectious individuals are thus required as they are efficient in preventing rapid transmission of COVID-19 [1, 4].

Among the various non-pharmaceutical control measures, contact tracing is widely adopted for combating COVID-19. Contact tracing refers to the process of identifying, evaluating, and managing individuals who have been exposed to those who have already proven to be infected with the COVID-19 virus [5]. It is effective for fighting the spread of the COVID-19 [6], but it usually requires labor-intensive efforts like interviewing the infected person and identifying their possible contacts. Digital contact tracing (DCT), based on information technologies such as mobile and biometric applications, can accelerate and improve the effectiveness of this contact tracing process [7]. Via DCT, people who may have come into close contact with a COVID-19 infected person can be electronically logged, tracked, contacted, and even isolated accordingly [8].

It is widely acknowledged that the benefits of DCT can be extensive and wide-reaching [4, 9, 10]. Based on their modelling, Ferretti et al. [10] suggest that an uptake rate of at least 56% would be sufficient to bring the reproduction rate under one and inhibit the spread of COVID-19. However, penetration rates in the majority of countries such as Germany, Australia, Switzerland, United Kingdom, France, or India are less than the suggestion of Ferretti et al. [10]. The best practice of how to enhance the acceptance rate is therefore an important issue for academics and practitioners. To date, an increasing number of studies have explored the factors influencing DCT acceptance. These empirical studies surely have added to the knowledge, but they have also produced inconsistent results. For example, several studies [11, 12] suggest a negative relationship between privacy concerns and one’s intention to use DCT or willingness to disclose personal information to DCT. Some evidence however has been found to the effect that privacy concerns have an insignificant effect on the intention to use DCT [13–18]. Mixed findings may cause confusion among the academics and the practitioners, and will thus be of limited assistance to fight in the spread of COVID-19.

To clarify the factors that facilitate DCT acceptance, several studies have begun to review related studies. For example, Megnin-Viggars et al. [19] conducted a rapid review regarding public’s engagement with DCT. Eleven studies have identified four themes of facilitators and five themes of barriers. Zetterholm et al. [20] undertook a scoping review with 25 studies and found that public acceptance varies across national cultures and sociodemographic strata. Furthermore, Zetterholm et al. [20] note that misconceptions about DCT and intention-action gap are topics in need of more resja-ch. In their systematic review, [18] identified 13 articles, which may positively or negatively influence the adoption of DCT. These reviews surely have improved our understanding about the acceptance of DCT. These studies however lack statistical synthesis of the reviewed data and therefore, a meta-analysis which statistically combines conceptually similar studies [21] and produces more objective evidence is requisite.

Considering the potential of DCT in future pandemic control and a better understanding of DCT acceptance, this study set out to perform a systematic review and meta-analysis to investigate the following research questions: (1) What is the current status of DCT acceptance research?; (2) What theories have been used to investigate DCT acceptance?; (3) What are the important antecedents that influence the acceptance of DCT?; and, (4) Does culture moderate the relationships between these antecedents and DCT acceptance?

## Methods

This study was conducted in conformity with the Preferred Reporting Items for a Systematic Review and Meta-analysis statement (PRISMA) [22] (*see* Supplementary file A). The Institutional Review Board of E-Da Hospital (EMRP-111-087) has approved the study protocol.

### Data sources and search strategy

Possibly related studies were identified by searching electronic databases, including Scopus, ScienceDirect, Springer, Wiley, Emerald, Taylor & Francis, and Dimensions until June 26th, 2023. Search terms combinations including COVID-19, contact tracing, and contact tracking were used. Detailed search queries for different databases are shown in Table 1.

**Table 1:** Search strategies for databases

Databases	Search terms
Scopus	TITLE-ABS-KEY (“COVID-19” AND (“contact tracing” OR “contact tracking”))
ScienceDirect	Title, abstract, keywords: “COVID-19” AND (“contact tracing” OR “contact tracking”)
Springer	“COVID-19” AND (“contact tracing” OR “contact tracking”)
Wiley	“COVID-19” AND (“contact tracing” OR “contact tracking”)
Emerald	“COVID-19” AND (“contact tracing” OR “contact tracking”)
Taylor & Francis	“COVID-19” AND (“contact tracing” OR “contact tracking”)
Dimensions	“COVID-19” AND (“contact tracing” OR “contact tracking”)

### Eligibility criteria and study selection

Studies that fulfilled the following criteria were selected: (1) Studies must have empirically investigated the acceptance of DCT with a quantitative or mixed approach; (2) Studies should have leverage theories as research underpinnings; and, (3) Studies selected from the literature must have been written in English and then peer-reviewed. Studies meeting the following criteria were excluded: (1) Studies that were conceptual or descriptive; (2) Studies were entirely qualitative in nature; or, (3) Studies where full texts were unavailable.

**Fig. 1:**
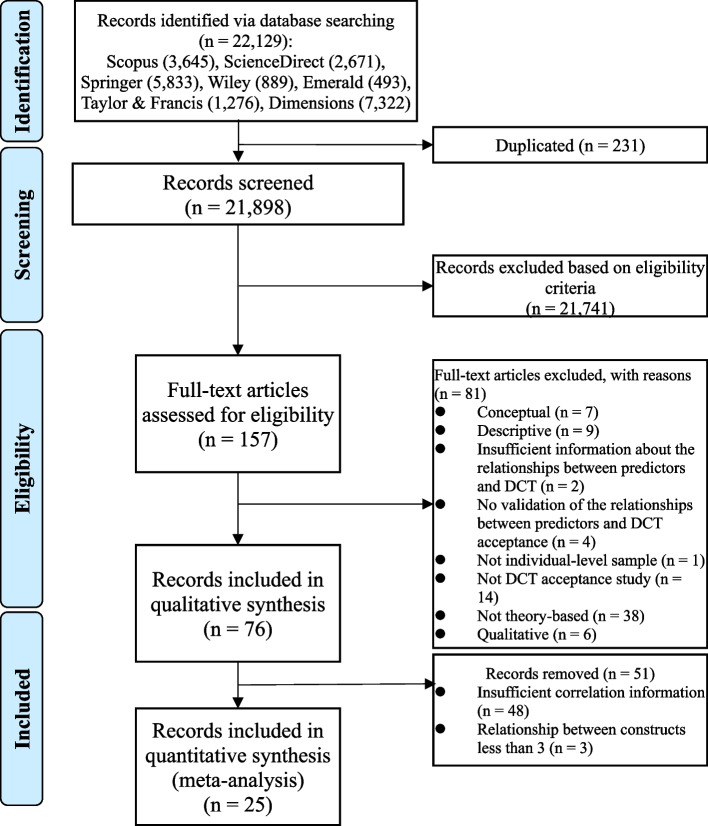
PRISMA flow diagram

In accordance with the stated inclusion and exclusion criteria, the studies were first assessed by the author and cross-checked by a colleague. For discrepancies that could not be resolved, a consensus meeting was held to ensure database accuracy. Prior evidence [23, 24] suggested that a given relationship between constructs should be covered at least three times to be included in a meta-analysis. As a result, three studies [25–27] were therefore excluded from the meta-analysis. Finally, the search generated 76 articles for systematic review and 25 articles for meta-analysis (*see* Fig. 1). Included articles for systematic review and meta-analysis were shown in Supplementary file B and C, respectively.

### Study quality assessment

Study quality was assessed in accordance with a quality assessment checklist for survey studies in psychology [28]. Mean overall quality score (*M* = 74.60, *SD* = 0.06) was close to the suggested threshold 0.75 [28]. Besides 15 studies [25, 29–42], other included studies [3, 9, 11, 13–17, 26, 27, 43–93] failed to report the justification of the sample size provided.

### Data extraction

For each included study, the following information was extracted: author name, publication year, correlation coefficient, sample size, the geographic area that a study covered, research methods (survey, experiment, …), and type of research (qualitative or mixed). Further, nomenclature used for antecedents were not entirely consistent across included studies, so that some of the antecedents that were adopted had similar meanings but were described in different words. For instance, perceived usefulness [14, 59] and performance expectancy [33, 34, 92] mean the same thing but wordings of the two are different. Such antecedents were combined under a unified name and the same words are purposefully adopted throughout the entire study.

### Data analytic procedures

A descriptive statistical analysis was first conducted to profile the characteristics of included studies. A systematic review and meta-analysis was then conducted to summarize the existing evidence of DCT acceptance and also to pool mean effect sizes (correlation correlations) of the relationships between antecedents and DCT acceptance. Fisher’s *Z* transformation was first used to transform correlation coefficients prior to conducting meta-analysis [21]. Mean effect sizes were calculated based on the inverse variance method weighted with sample sizes. Furthermore, 95% confidence interval, 95% prediction interval, *Q*, *I* ^*2*^, and *H* index were then derived. Publication bias was assessed by using fail-safe N [94].

## Results

### Study characteristics

Table 2 summarizes the attributes of the 76 primary studies included in this study. In these included studies, the sample size ranged from 137 to 9555 with a mean of 951.33 and a standard deviation of 1203. The number of publications rose from 6 to 2020 to 32 in 2022, representing a more than 500% increase. Half of included studies were conducted in Europe. Most studies adopted survey as their primary research method (96.05%) and are quantitative in nature (94.74%).

**Table 2:** Characteristics of eligible studies

Characteristics		Frequency	%
Sample size	M (SD) = 951.33 (1203)	Min = 137	Max = 9555
Publication year	2020	6	7.89%
	2021	32	42.11%
	2022	31	40.79%
	2023	7	9.21%
Area	Europe	38	50.00%
	Asia	20	26.32%
	Americas	9	11.84%
	Oceania	6	7.89%
	Africa	3	3.95%
Methods	Survey	73	96.05%
	Experiment	2	2.63%
	Design Science	1	1.32%
Type of evidence	Quantitative	72	94.74%
	Qualitative + Quantitative	3	3.95%
	Qualitative	1	1.32%

*M* means mean, *SD* denotes standard deviation, *Min* means minimum value and *Max* denotes maximum value

#### Antecedents of DCT acceptance

**Fig. 2:**
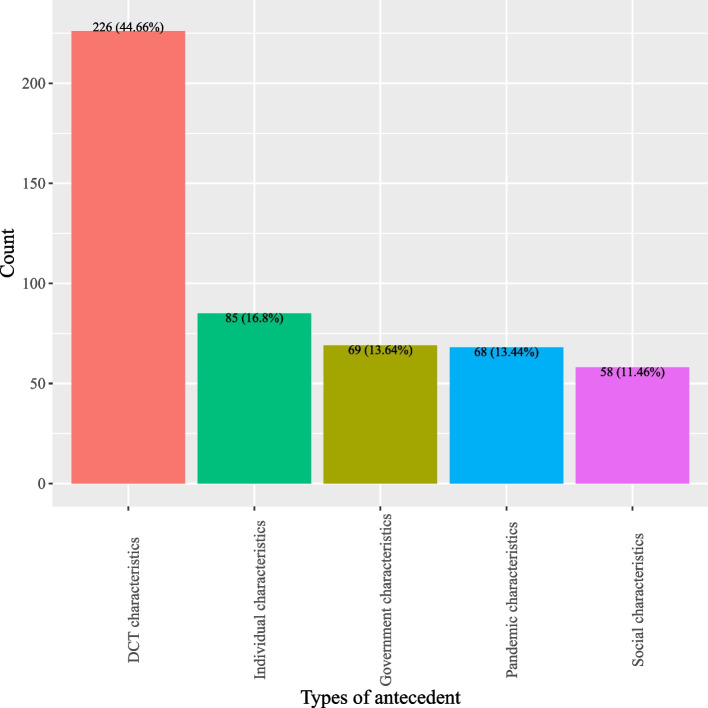
Distribution of antecedent types

**Fig. 3:**
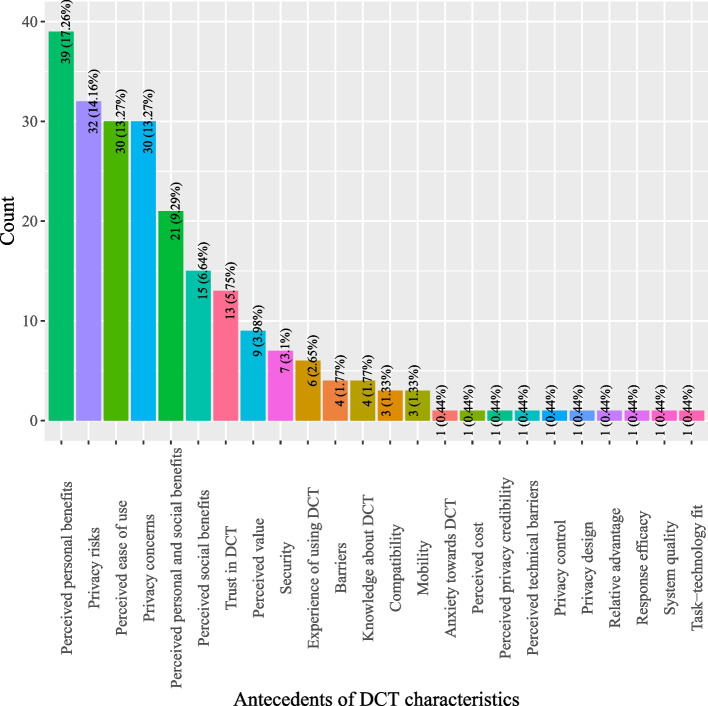
Distribution of antecedents of DCT characteristics

**Fig. 4:**
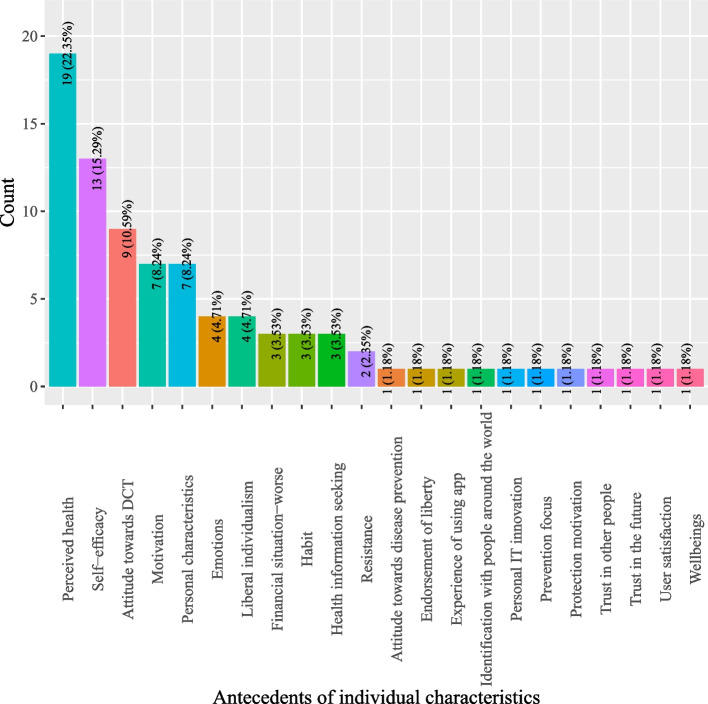
Distribution of antecedents of individual characteristics

**Fig. 5:**
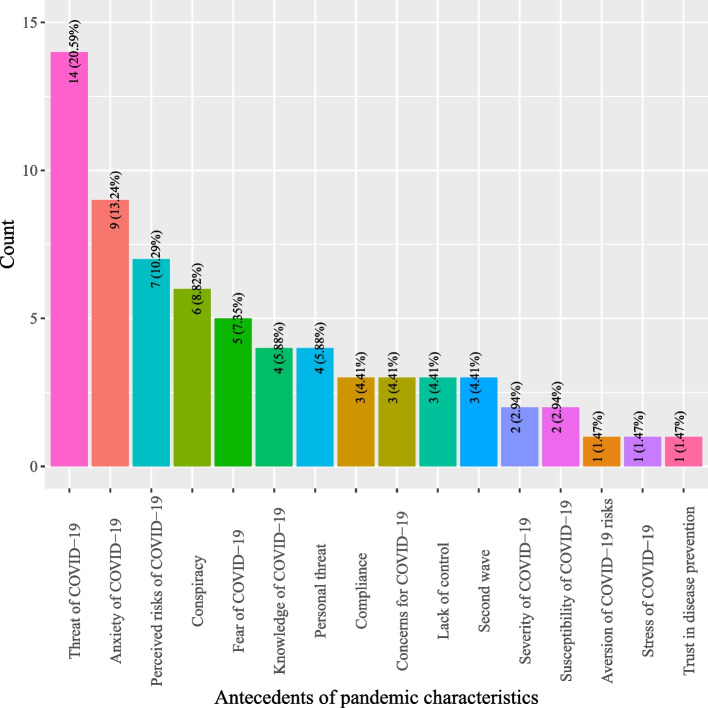
Distribution of antecedents of pandemic characteristics

**Fig. 6:**
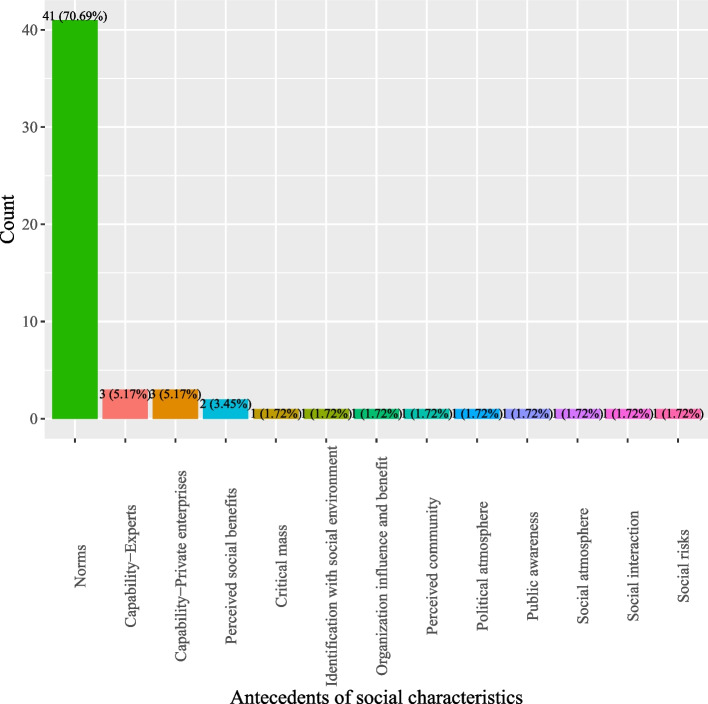
Distribution of antecedents of social characteristics

**Fig. 7:**
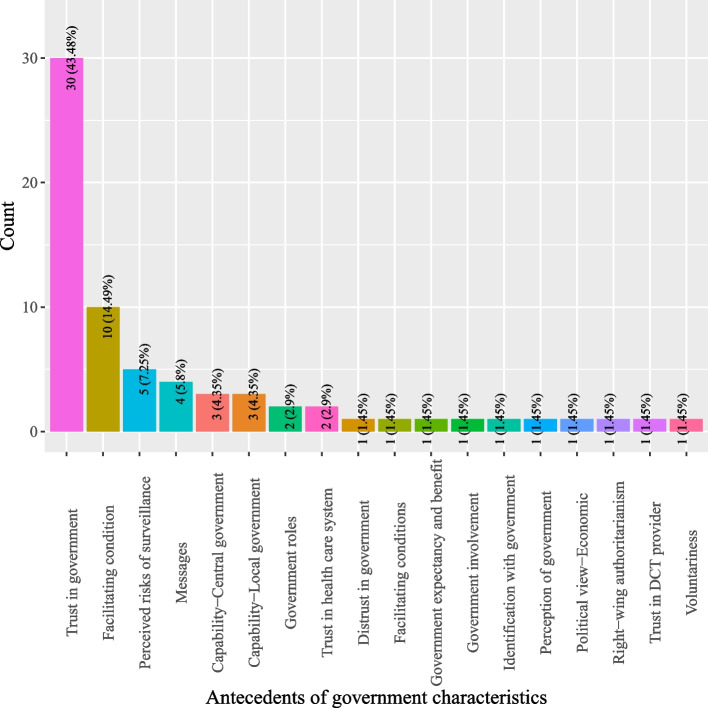
Distribution of antecedents of government characteristics

Based on definitions of antecedents for DCT acceptance, this study categorized antecedents into five classes: (1) DCT characteristics; (2) individual characteristics; (3) pandemic characteristics; (4) social characteristics; and, (5) government characteristics. As shown in Fig. 2, most studies investigated antecedents pertinent to DCT (44.66%) which describe the characteristics of DCT, such as perceived personal benefits, privacy risks, perceived ease of use, and privacy concerns (*see* Fig. 3). Individual characteristics that delineate factors related to users of DCT were the second most studied antecedents (16.8%) (*see* Fig. 2). Often studied antecedents included perceived health status, self-efficacy and attitude toward DCT (*see* Fig. 4). Around 11.46% of the studies examined antecedents relevant to characteristics of the society as a whole (*see* Fig. 2). Norms (e.g., social influence or subjective norms) and capabilities (experts and private enterprises) were often researched antecedents (*see* Fig. 5). Approximately 13.44% of the studies examined characteristics that describe the pandemic (*see* Fig. 2), antecedents such as threat of COVID-19, anxiety of COVID-19, perceived risks of COVID-19, conspiracy, and fear of COVID-19 were often examined (*see* Fig. 6). Finally, about 13.64% of the studies scrutinized characteristics that related to governments, frequently examined antecedents including trust in government, facilitating conditions, perceived risks of surveillance and capabilities (central government and local government) (*see* Fig. 7).

### Theories used for studying DCT acceptance

A total of 38 different theories/models/frameworks pertinent to 8 disciplines have been adopted 115 times in the selected studies (*see* Table 3). Over 47% of the adopted theories belong to information systems (IS). In IS discipline, technology acceptance model (TAM) [95] and unified theory of acceptance and use of technology (UTAUT) [96] are the two most cited theories (14, 25.45% in IS discipline, respectively), followed by privacy calculus theory [97, 98] (13, 23.64%). Theories from public health and psychology are the second and third most adopted for investigating DCT acceptance (24, 20.87% vs. 16, 13.91%). Health belief model [99] and protection motivation model [100] are most chosen in public health (13, 54.17% in public health vs. 10, 41.67%), while theory of planned behavior [101] and theory of reasoned action [102] are most used (4, 25% vs. 3, 18.75%) in psychology. The remaining theories are from sociology (11, 9.57%), communication (3, 2.61%), marketing (3, 2.61%), politics (2, 1.74%), and law (1, 0.87%). Detailed information about theories used are shown in Table 3.

**Table 3:** Theories used in DCT acceptance studies

Discipline (n, %)	Theory	FREQ	%
Information systems (55, 47.83)	Technology acceptance model	14	25.45%
	Unified theory of acceptance and use of technology	14	25.45%
	Privacy calculus theory	13	23.64%
	Technology acceptance model 2	4	7.27%
	APCO model	3	5.45%
	Unified theory of acceptance and use of technology 2	3	5.45%
	Expectation confirmation theory	1	1.82%
	Health information technology acceptance model	1	1.82%
	Internet users’ information privacy concern	1	1.82%
	Task-technology fit	1	1.82%
Public health (24, 20.87)	Health belief model	13	54.17%
	Protection motivation theory	10	41.67%
	Risk-risk tradeoff	1	4.17%
Psychology (16, 13.91)	Theory of planned behavior	4	25.00%
	Theory of reasoned action	3	18.75%
	Cognitive appraisal theory	1	6.25%
	Compensatory control model	1	6.25%
	Crisis decision theory	1	6.25%
	Hofstede’s cultural dimension theory	1	6.25%
	Psychological reactance theory	1	6.25%
	Regulatory focus theory	1	6.25%
	Risk calculus theory	1	6.25%
	Self-determination theory	1	6.25%
	Theory of valuation	1	6.25%
Sociology (11, 9.57)	Privacy theory	3	27.27%
	Trust theory	3	27.27%
	Social exchange theory	2	18.18%
	Social identify theory	1	9.09%
	Social norm	1	9.09%
	Theory of distrust	1	9.09%
Communication (3, 2.61)	Diffusion innovation theory	1	33.33%
	Theory of normative social behavior	1	33.33%
	Uncertainty reduction theory	1	33.33%
Marketing (3, 2.61)	Theory of consumption values	2	66.67%
	Theory of innovation resistance	1	33.33%
Politics (2, 1.74)	Social conservatism	1	50.00%
	Social contract theory	1	50.00%
Law (1, 0.87)	Procedural fairness theory	1	100.00%

*FREQ* denotes frequency, *APCO* means antecedents-privacy concerns-outcomes

### DCT acceptance intention/behavior

In the included studies, various intentions/behaviors have been adopted to investigate DCT acceptance (*see* Table 4). This study classified differing intentions/behaviors into four types: intention to use, intention to recommend, actual use, and continuance intention. To examine DCT acceptance, intention to use was the most adopted (82.72%). Actual use (9.88%), continuance intention (6.17%), and intention to recommend (1.23%) was used least.

**Table 4:** Intention/behavior of DCT acceptance

Type (n, %)	Intention/behavior	FREQ	%
Intention to use (67, 82.72)	Intention to use	45	67.16%
	Intention to adopt	10	14.93%
	Behavioral intention	3	4.48%
	Intention to download	2	2.99%
	Intention to install	2	2.99%
	Acceptance	1	1.49%
	Willingness to disclose	1	1.49%
	Willingness to disclose data	1	1.49%
	Willingness to disclose personal information	1	1.49%
	Willingness to falsify personal information	1	1.49%
Intention to recommend (1, 1.23)	Intention to recommend	1	100.00%
Actual use (8, 9.88)	Actual use	8	100.00%
Continuance intention (5, 6.17)	Continuance intention	4	80.00%
	Intention to continue use	1	20.00%

*FREQ* denotes frequency

### Meta-analysis results

Twenty-five studies with 14 relationships were finally included for subsequent meta-analysis. This study first assessed the extent of heterogeneity of effect sizes based on *Q*, *I* ^*2*^, and *H* index [21, 103, 104]. A significant *p* value of *Q* test [21], an *I* ^*2*^ value > 25% [104], or the lower limit of 95% confidence interval of *H* greater than 1 [103] are an indication of the presence of heterogeneity. As depicted in Table 5, most studies investigating relationships revealed different degrees of heterogeneity. As a result, the random effects model was therefore adopted for pooling summary effects [21]. Forest plots of investigated relationships may be found in Supplementary file D.

Table 6 shows the results of meta-analysis of the relationships between antecedents and DCT acceptance. An examination at the 95% confidence intervals of investigated relationships demonstrated that 12 out of 14 relationships were significant because zero was not contained in these 95% confidence intervals [105]. Insignificant antecedents of DCT acceptance included privacy concerns and fear of COVID-19. Among the 12 significant relationships, privacy risks correlated with intention to use in a negative direction while other constructs correlated with DCT acceptance in a positive direction. The summary effect size of perceived social benefits is the largest (0.67), while identification with social environment has the smallest mean effect size (0.08). Furthermore, the 95% prediction intervals of most relationships show a wider range than their respective 95% confidence intervals, signifying the presence of heterogeneity [106], further supporting the use of random effects models.

To diminish the possibility of publication bias, this study searched multiple databases. Further, fail-safe N was employed to assess possible publication bias [94]. A rule of thumb is that the fail-safe N should reflect no less than five times the number of studies included in the meta-analysis plus 10 [94]. As shown in Table 6, all relationships exceed Rosenthal’s rule of thumb except for the relationship between identification with social environment and intention to use.

**Table 5:** The results of heterogeneity tests

Antecedents	Intention	k	*N*	*Q*	*p* value	*I* ^*2*^	95% C.I.		*H*	95% C.I.	
Perceived personal benefits	Intention to use	11	8803	916.46	0.000	0.99	0.99	0.99	9.57	8.53	10.74
Privacy concerns	Intention to use	9	7452	418.93	0.000	0.98	0.97	0.99	7.24	6.20	8.45
Perceived ease of use	Intention to use	8	3002	361.52	0.000	0.98	0.97	0.99	7.19	6.09	8.48
Perceived social benefits	Intention to use	6	5014	541.78	0.000	0.99	0.99	0.99	10.41	8.93	12.14
Trust in DCT	Intention to use	4	5119	1039.24	0.000	1.00	1.00	1.00	18.61	16.31	21.24
Privacy risks	Intention to use	3	1022	24.63	0.000	0.92	0.79	0.97	3.51	2.20	5.59
Attitude towards DCT	Intention to use	5	2814	428.11	0.000	0.99	0.99	0.99	10.35	8.71	12.29
Self-efficacy	Intention to use	4	2687	17.95	0.000	0.83	0.57	0.93	2.45	1.53	3.90
Fear of COVID-19	Intention to use	3	2226	81.46	0.000	0.98	0.95	0.99	6.38	4.59	8.87
Norms	Intention to use	10	4278	172.75	0.000	0.95	0.92	0.97	4.38	3.59	5.35
Identification with social environment	Intention to use	3	1044	0.07	0.966	0.00	0.00	0.90	1.00	1.00	3.10
Trust in the government	Intention to use	5	4513	58.08	0.000	0.93	0.87	0.96	3.81	2.76	5.26
Facilitating conditions	Intention to use	3	858	63.33	0.000	0.97	0.94	0.98	5.63	3.94	8.03
Identification with the government members	Intention to use	3	1044	0.33	0.848	0.00	0.00	0.90	1.00	1.00	3.10

*k* means number of studies, *N* means total sample size and *C.I.* denotes confidence interval

**Table 6:** Meta-analysis results

Characteristics	Antecedents	Intention	k	N	Mean E.S.	95% C.I.		95% P.I.		Fail-safe N
DCT	Perceived personal benefits	Intention to use	11	8803	0.53	0.36	0.67	-0.24	0.89	9237
	Privacy concerns	Intention to use	9	7452	-0.16	-0.35	0.04	-0.73	0.54	589
	Perceived ease of use	Intention to use	8	3002	0.44	0.18	0.64	-0.53	0.91	1736
	Perceived social benefits	Intention to use	6	5014	0.67	0.45	0.82	-0.39	0.97	7371
	Trust in DCT	Intention to use	4	5119	0.63	0.29	0.83	-0.89	0.99	2690
	Privacy risks	Intention to use	3	1022	-0.26	-0.46	-0.04	-1.00	0.99	78
Individual	Attitude towards DCT	Intention to use	5	2814	0.59	0.28	0.79	-0.69	0.98	2154
	Self-efficacy	Intention to use	4	2687	0.27	0.16	0.37	-0.23	0.65	261
Pandemic	Fear of COVID-19	Intention to use	3	2226	0.09	-0.29	0.45	-1.00	1.00	65
Social	Norms	Intention to use	10	4278	0.57	0.47	0.66	0.11	0.83	6701
	Identification with social environment	Intention to use	3	1044	0.08	0.02	0.14	-0.30	0.44	5
Government	Trust in the government	Intention to use	5	4513	0.42	0.33	0.51	0.05	0.69	1157
	Facilitating conditions	Intention to use	3	858	0.61	0.33	0.79	-1.00	1.00	446
	Identification with the government members	Intention to use	3	1044	0.24	0.18	0.30	-0.15	0.57	66

*DCT* means digital contact tracing, *k* denotes number of studies, *N* means total sample size, *E.S.* refers to effect size, *C.I.* denotes confidence interval and *P.I.* means prediction interval

### Subgroup analysis results

In order to account for the heterogeneity identified in this study, several subgroup analyses were conducted. Since the relationships between constructs have to be divided into two groups based on the scores of individualism/collectivism or uncertainty avoidance. Only the relationships that were examined at least two times in each subgroup were included in this analysis. Since COVID-19 is a worldwide issue, this study considered culture an important moderator for DCT acceptance. Based on the cultural dimensions of Hofstede et al. [107], dimensions including individualism/collectivism, measuring the degree of an individual’s independence/dependence on groups [107], and uncertainty avoidance, referring the extent to which individuals in a society feel threatened by uncertain and equivocal situations [107], were used as the grouping variables. Individualism/collectivism and uncertainty avoidance were chosen since using DCT relates to every individual’s personal decision. COVID-19 is widely considered a critical threat to our society and to the future, which reflects both the characteristics of individualism/collectivism and uncertainty avoidance.

This study divided the included studies into low- and high-score groups based on mean of individualism/collectivism and uncertainty avoidance scores. A subgroup analysis, based on individualism/collectivism scores, showed a higher mean effect size, for the relationship between perceived personal benefits and intention to use, for low-score group (0.65) than that of high-score group (0.41), but this did not have statistical significance (*p* = 0.093). The relationship between norms and intention to use showed a significant higher mean effect size for high-score group (0.68) than that of low-score group (0.49). For the relationship between privacy concerns and intention to use, the high-score group (-0.28) had a higher mean effect size than the low-score group (0.10) and the difference was significant (*p* = 0.040). The relationship between perceived ease of use and intention to use showed a non-significant higher mean effect size for the high-score group (0.52) than that of the low-score group (0.39). The relationship between attitude towards DCT and intention to use showed a similar result as the relationship between perceived ease of use and intention to use (*see* Table 7).

**Table 7:** Subgroup analysis based on individualism/collectivism

Path	IND	k	N	Mean E.S.	95% C.I.		*I* ^*2*^	95% C.I.		*p* value
Perceived personal benefits◊Intention to use	Low	5	2342	0.65	0.45	0.79	0.95	0.92	0.97	0.093
	High	6	6461	0.41	0.18	0.61	0.99	0.98	0.99	
Norms◊Intention to use	Low	6	2212	0.49	0.37	0.59	0.95	0.91	0.97	0.016
	High	4	2066	0.68	0.57	0.76	0.80	0.48	0.92	
Privacy concerns◊Intention to use	Low	3	1482	0.10	-0.20	0.38	0.95	0.90	0.98	0.040
	High	6	5970	-0.28	-0.46	-0.08	0.99	0.98	0.99	
Perceived ease of use◊Intention to use	Low	5	1487	0.39	0.03	0.66	0.99	0.98	0.99	0.623
	High	3	1515	0.52	0.07	0.79	0.96	0.91	0.98	
Attitude towards DCT◊Intention to use	Low	2	587	0.47	-0.16	0.83	0.98	0.95	0.99	0.530
	High	3	2227	0.66	0.24	0.87	0.99	0.99	1.00	

*IND* denotes individualism/collectivism, *k* means number of studies, *N* means total sample size, *E.S.* refers to effect size, *C.I.* means confidence interval and *NA* refers to not available

Regarding the dimension of uncertainty avoidance, the relationships of intention to use with perceived personal benefits, perceived ease of use, and perceived social benefits all revealed a similar pattern. This demonstrated that the low-score group had a higher mean effect size than that of low-score group, while the associations of intention to use with norms, privacy concerns, and attitude towards DCT showed a reverse result. No relationships in this subgroup analysis reached statistical significance (*see* Table 8).

**Table 8:** Subgroup analysis based on uncertainty avoidance

Path	UA	k	N	Mean E.S.	95% C.I.		*I* ^*2*^	95% C.I.		*p* value
Perceived personal benefits◊Intention to use	Low	6	2579	0.58	0.36	0.74	0.98	0.97	0.99	0.463
	High	5	6224	0.46	0.18	0.68	0.99	0.99	1.00	
Norms◊Intention to use	Low	8	2926	0.55	0.42	0.65	0.95	0.93	0.97	0.322
	High	2	1352	0.66	0.44	0.80	0.59	0.00	0.90	
Privacy concerns◊Intention to use	Low	4	2412	0.00	-0.28	0.28	0.96	0.92	0.98	0.154
	High	5	5040	-0.28	-0.49	-0.03	0.99	0.98	0.99	
Perceived ease of use◊Intention to use	Low	6	1696	0.46	0.14	0.69	0.99	0.98	0.99	0.794
	High	2	1306	0.38	-0.21	0.77	0.85	0.41	0.96	
Perceived social benefits◊Intention to use	Low	2	1412	0.76	0.38	0.92	0.99	0.97	0.99	0.461
	High	4	3602	0.62	0.29	0.82	0.99	0.99	1.00	
Attitude towards DCT◊Intention to use	Low	3	1983	0.41	0.05	0.68	0.99	0.98	0.99	0.062
	High	2	831	0.77	0.50	0.91	0.98	0.95	0.99	

*UA* denotes uncertainty avoidance, *k* means number of studies, *N* means total sample size, *E.S.* refers to effect size, *C.I.* means confidence interval and *NA* refers to not available

## Discussion

Available reports regarding DCT acceptance from existing evidence were synthesized. Collected data were first systematically reviewed and then meta-analyzed. Among the five types of antecedents, DCT-related characteristics were the most examined antecedents of DCT acceptance. Perceived personal benefits and privacy risks related to DCT-related characteristics were examined by most studies as antecedents of DCT acceptance. Theories including TAM, UTAUT, and privacy calculus theory from the IS field and health belief model from the public health discipline were most-employed as theoretical underpinnings for investigating DCT acceptance. Further, intention to use was the most-used surrogate construct for DCT acceptance. Regarding results of meta-analysis, perceived social benefits was the most-influential and positive antecedents of DCT acceptance (mean effect size = 0.67), while privacy risks was a significant and negative antecedent of DCT acceptance (mean effect size = -0.26). Antecedents including privacy concerns and fear of COVID-19 were not significant predictors of DCT acceptance. Further, only individualism/collectivism of cultural dimensions [107] was a significant moderator for the relationships between norms and DCT acceptance and privacy concerns and DCT acceptance. Several important findings based on this systematic review and meta-analysis deserve further discussion.

Although Trang et al. [9] argued that DCT is characterized by unclear personal and social benefits, both perceived personal benefits and perceived social benefits were found to be a significant antecedent of DCT acceptance with a near strong mean effect size of 0.53 and 0.67, respectively. These findings may be explained by the notions of egoism (emphasizing the benefits for individual self) and altruism (highlighting the benefits for others) [108].

According to psychological egoism [109], every behavior and decision of individuals is driven by self-interest. However, promoting altruistic behaviors, including the acceptance of DCT, can require significant efforts [110]. While some may expect DCT to primarily benefit themselves by identifying potential infections, it is important to acknowledge that in the context of a global pandemic like COVID-19, the World Health Organization [111] and many countries have emphasized the use of DCT. This highlights the clear societal-level benefits of DCT as well.

Privacy concerns were not found to be a significant predictor of DCT acceptance, as indicated by this meta-analysis; however, privacy concerns have traditionally been recognized as a crucial focus in studies related to various information technologies. The number of privacy-related studies has increased significantly in recent years [112]. In many of these studies, concerns about privacy are shown to inhibit the intention to use an location-based service (LBS) in different domains [113, 114]. As such, the use of DCT may involve similarities with using LBS since users may need to disclose their location or personal information [115, 116]. One principal difference between an LBS and DCT is that a DCT may be pertinent to an individual’s health, while an LBS is not, which may lead to the insignificant findings. In fact, privacy concerns have produced inconsistent or even contradictory results in a DCT usage context of DCT usage [13–16, 53].

This study found that perceived ease of use significantly predicts DCT acceptance. This aligns with the results of a previous meta-analyses on the TAM [117] and the underlying principles of the model itself [118]. According to TAM, if the use of DCT becomes complicated and demands mental or physical effort, individuals may be less inclined to adopt it [118].

Trust in DCT, based on meta-analysis results, was found to be a significant predictor of DCT acceptance in this study. Trust is usually defined as an individual’s (trustors) willingness to depend on another party (trustees) due to the other party’s given characteristics [76]. Trustors however may face subsequent risk and uncertainty when they rely on trustees to complete their tasks [119]. In the context of DCT, the trustee is now represented as a technological entity rather than as a person, though there may still be some level of risk and uncertainty. Since the rationale behind DCT is clear and easy to understand, most users tend to trust its use.

Privacy risks were found to be a significant and negative predictor of DCT acceptance, with a mean effect size of -0.26, indicating a small-to-medium impact. Privacy risks differ from privacy concerns, which primarily involve individuals’ worries regarding potential opportunistic practices related to their personal information [120]. Privacy risks, on the other hand, encompass individuals’ awareness of uncertainty and the potential negative consequences of sharing personal information with others [121]. In a meta-analysis examining the effects of privacy concerns and privacy risks on the intention to disclose personal information, Yu et al. [122] found that privacy risks had a substantial impact on disclosure intention and behavior, while privacy concerns only had a minor influence. This finding is consistent with the results reported by Yu et al. [122] and supports the significance of privacy risks in relation to DCT acceptance.

One’s attitude towards DCT was confirmed to be a significant and positive predictor of DCT acceptance with a mean effect size of 0.62. Generally speaking, public opinion related to DCT has an acceptance rate between 40% and 60% as favored worldwide [63]. This result corroborates the findings of another meta-analysis study [123] that attitude, based on theory of planned behavior [101], is a near strong predictor of intention.

This study confirmed self-efficacy or perceived behavioral control as a significant and positive predictor of DCT acceptance with a near moderate mean effect size of 0.27. This finding is consistent with a meta-analysis [124] reporting self-efficacy as a significant predictor of health-related intentions and behavior. This confirms the notion of theory of planned behavior [101] regarding the association between perceived behavioral control and behavioral intention.

Although individuals naturally experience fear when confronted with a contagious disease that threatens their well-being or even mortality, fear of COVID-19 was not identified as a significant predictor of DCT acceptance in this study. One potential explanation for this lack of significance could be the asymmetric nature of the threat posed by COVID-19 [38]. Older or chronically ill individuals are more susceptible to the risks associated with COVID-19 when compared to younger and healthier individuals [125]. The participants included in the studies encompassed a specified range of health and age profiles, which might have influenced this particular finding.

Norms such as social influence were found to have a significant and medium-to-large (mean effect size = 0.57) relationship with DCT acceptance, according to this meta-analysis. This finding reflects the perspective of theory of planned behavior [101], and also the finding of a meta-analysis of the impact of norms on health-related intentions and behaviors [124].

Identification with the social environment and identification with government members were both found to be significant and positive predictors of DCT acceptance in this study. In fact, the reason why identification is a significant predictor of DCT acceptance may be based on social identity theory [126]. This notion considers that we are all embedded in complicated social structures, ranging from national identity to social groups that may shape much of our behavior. As such, people may choose to identify with those who benefit from DCT and/or those who recommend using it [80]. However, the use of fail-safe N to establish the relationship between identification with government members may suffer from publication bias [94].

Trust in the government, in the current study, was found to be both a positive and significant antecedent of DCT acceptance with a near medium effect (0.42). Government is considered as a relatively small group of people who are elected to represent constituents. If the government handles the COVID-19 pandemic appropriately, people should have faith in their responsible government. As such, DCT applications that were government-provided or accredited should be nominally accepted due to peoples’ stated trust in their government representatives.

It is evident that the utilization of DCT necessitates the presence of organizational and technological infrastructure to provide critical support [127]. Without these supportive resources, individuals are unable to utilize DCT effectively with any degree of trust. The meta-analysis results confirm that facilitating conditions play a significant role in predicting DCT acceptance. This finding aligns with the principles of the UTAUT 2 [128], which asserts that facilitating conditions influence an individual’s intention to use information technologies.

Significant statistical differences were identified in the subgroup analysis based on Hofstede et al. [107]’s individualism/collectivism dimension. This study observes that the relationship between norms and acceptance of DCT is stronger in individualistic countries when compared to collectivist or low individualistic countries. This finding contradicts the notion that there is greater pressure to conform to social norms in collectivist cultures as compared to individualistic countries [107]. Interestingly, a study by Vishkin et al. [129] discovered that adherence to emotional norms is stronger in individualistic cultures than in collectivist ones. This suggests that different types of norms have varying effects on individuals’ conformity to follow norms. Given that COVID-19 is unprecedented as a pandemic that most individuals have not previously encountered, it remains uncertain how individuals’ norms are being influenced or how new norms are developing, which could contribute to this particular finding.

Additionally, the association between privacy concerns and DCT acceptance was significantly stronger in higher individualistic countries when compared to those with lower individualistic tendencies. This finding aligns with existing evidence [130] that individuals in higher individualistic countries tend to exhibit higher levels of privacy concerns than individuals in lower individualistic countries.

### Theoretical implications

Based on the findings of this review, several theoretical implications can be derived. First, on scrutinizing the antecedents of DCT acceptance, this review found much emphasis placed on those factors pertaining to DCT characteristics. Despite such endeavors, antecedents such as privacy concerns and fear of COVID-19 are not able to predict DCT acceptance. Further research remains to explore other salient antecedents of DCT acceptance based on this perspective. Furthermore, important factors pertinent to individual, social, governmental, and the healthcare community’s actual response to the COVID-19 pandemic must remain at the forefront since DCT will not work effectively if only technological issues are considered. Secondly, the technology acceptance model, unified theory of acceptance and use of technology, and privacy calculus theory, as based on the findings of this study, have proven to be useful theories to explain DCT acceptance. Beneficial factors including perceived personal benefits, perceived social benefits, and risk factors including privacy risks are significant predictors of DCT acceptance.

Despite perceived personal benefits and perceived social benefits being both significant predictors of DCT acceptance, these two benefits may not easy to distinguish apart. In the study of Abramova et al. [3], they treat both constructs as a single construct “benefits” because participants cannot distinguish appropriately between the two benefits. Future research should further clarify salient differences in these two benefits. This is important because the effect of perceived social benefits on DCT acceptance is among the highest determinant of DCT acceptance. This issue may influence how healthcare authorities formulate the best strategies and practices for promoting the acceptance of DCT. Thirdly, confirming the critical role of antecedents of DCT acceptance, the next reasonable step is to examine important factors that may influence these identified antecedents. By doing so, a more holistic DCT acceptance model can be reasonably proposed.

### Practical implications

Several practical implications for expanding DCT acceptance are obtainable based on these results. First, DCT should include privacy protection as one of the key design features. For instance, the developer could incorporate visual cues within the system design that enhance individuals’ awareness of privacy protection, with the goal of building trust among users. When considering designs, convenience should be a priority. This includes making them simple and easy to use. The personal and social benefits that DCT provides are clearly explained, thus aiming to foster concerned individuals’ positive attitudes towards DCT. When promoting DCT acceptance, sufficient training programs should be provided in order to enhance individuals’ self-efficacy towards DCT usage. The influence of significant-others cannot, and should not, be neglected. More awareness about social norms can be generated by organizing workshops related to DCT, with enthusiastic DCT-users invited to share their personal experiences and a shared sense of security. Appropriate strategies for fostering social and national identity should be proposed aiming to shape individuals’ attitudes towards DCT acceptance on a localized-basis. Finally, governments should endeavor to fight the COVID-19 pandemic, or any other emerging infectious diseases for that matter, in order to strengthen individuals’ faith or trust in them. When individuals trust their governments, they follow governmental instructions about promoting the use of DCT.

### Directions for further research

Understanding our past is crucial, but of equal importance is the charting our future course. Beneath this study lies a list of unresolved questions that could illuminate our comprehension of factors influencing the acceptance of DCT.

#### Lack of a comprehensive model for predicting the acceptance of DCT

It is clear from the results of this review that diversified theories from differing disciplines have been adopted to tackle this issue. These theories have provided important perspectives for understanding factors that influence the acceptance of DCT. What is missing is a unified theory for explaining the acceptance of DCT. This unified theory should not be focused from a technical perspective alone, while ignoring other perspectives such as public health, psychology, sociology, communication, or marketing. With such a unified view, managers can use this tool to understand the drivers of acceptance in order to deploy proactive interventions aimed at improving the DCT acceptance rate. As a result, researchers may wish to compare different theories or models and subsequently develop a unified model that synthesizes elements from these available theories or models.

#### How perceived personal and social benefits work?

Based on the results of this review, perceived personal benefits and social benefits emerge as significant predictors of DCT acceptance, with perceived social benefits identified as the most important motivator of all. Now, pivotal questions continue to revolve around an understanding of how these two categories of benefits influence individuals’ acceptance of DCT. As previously discussed, it is imperative to delineate the distinctions between these two benefit types. With a clearer grasp of this differentiation, managers can produce targeted interventions aimed at enhancing DCT acceptance by underscoring the relative significance of both personal and social benefits. Researchers can adopt the perspectives of psychological egoism and altruism to achieve this goal. Specifically, measures for personal and social benefits should be crafted to ensure a clear distinction between given benefits. This will effectively dismiss any existing confusion surrounding and between perceived personal and social benefits.

#### Do privacy concerns really fail to matter?

Privacy concerns have been established as an important factor inhibiting individuals from engaging in specific behaviors across a variety of disciplines [112]. However, this review was unable to uncover conclusive evidence supporting the notion that privacy concerns consistently act as a barrier to the acceptance of DCT. This suggests that there is still ample opportunity for the exploration into the true impact of privacy concerns on DCT acceptance. A more comprehensive understanding of how privacy concerns influence individuals can aid managers in developing effective strategies to enhance DCT acceptance. In the exploring this issue, researchers may choose to adopt a perspective regarding the privacy paradox [131] to address existing ambiguities. The study conducted by Yu et al. [122] provides valuable insights into this matter. Yu et al. [122] found that perceived privacy risks can significantly reduce individuals’ intention to disclose personal information, in addition to their actual information disclosure behavior. However, privacy concerns appear to primarily affect disclosure intention and have a limited impact on actual information disclosure behavior. Notably, only a few studies [58, 59] reviewed in this study have simultaneously examined these two constructs as part of their models. By doing so, the relationship between privacy concerns and the acceptance of DCT may yet be clarified.

#### What are the key factors that influence the antecedents identified in this review?

Based on the findings of this review, we have identified important antecedents that predict DCT acceptance. The key question now is how to effectively manipulate these antecedents in order to enhance DCT acceptance. One potential solution is to further explore the factors that impact these antecedents. By doing so, managers can adjust these influencing factors to positively affect the antecedents we have identified in this review, consequently leading to improved DCT acceptance. Moreover, by incorporating the factors that may influence these identified antecedents, we can gain a deeper understanding of the relationships between these antecedents and DCT acceptance. The decomposed theory of planned behavior has served to illustrate this concept [132].

### Limitations

Several limitations are notable in the present study. First, studies were extracted from certain popular electronic databases; meaning, some relevant articles may not have been extracted. Future review studies may consider a wider range of electronic databases for purposes of extraction. Second, only quantitative or mixed studies were included which may lead to sampling bias. Thirdly, this study only validates relationships separately, so future studies may require testing the relationships by using meta-analysis structural equation modeling techniques.

## Conclusion

This study synthesizes the results from prior studies on DCT acceptance by using systematic review and meta-analysis. Specifically, the current study examines the influence of factors related to DCT characteristics, individual characteristics, social characteristics, governmental characteristics, and pandemic characteristics. Perceived personal benefits, perceived ease of use, perceived social benefits, trust in DCT, privacy risks, attitudes towards DCT, self-efficacy, norms, identification with social environment, trust in the government, facilitating conditions, and identification with government members were found to be significant antecedents of DCT acceptance. Further, it was also found that one’s culture significantly moderates the relationships between personal norms/privacy concerns and individual intention to use DCT. Future research could further investigate antecedents based on the findings of this review. Furthermore, healthcare authorities and governments can foster more suitable strategies to promote DCT acceptance for confronting COVID-19, or other emergent infectious disease outbreaks.

### Supplementary Information

**Additional file 1: Supplementary file A.** PRISMA 2020 checklist. **Supplementary file B.** Summary of included studies for systematic review. **Supplementary file C.** Studies included in meta-analysis. **Supplementary file D.** Forest plots

## Data Availability

The datasets used and analyzed during the current study are available from the corresponding author upon reasonable request.

## Abbreviations

CI: Confidence interval
COVID-19: Coronavirus disease 2019
DCT: Digital contact tracing
ES: Effect size
IND: Individualism/collectivism
M: Mean
PI: Prediction interval
SARS: Severe acute respiratory syndrome
SD: Standard deviation
UA: Uncertainty avoidance
