# Supplementary material for: Antecedents predicting digital contact tracing acceptance: a systematic review and meta-analysis
Source: BMC Med Inform Decis Mak. 2023 Oct 11;23:212. doi: 10.1186/s12911-023-02313-1 (PMC10568897; doi:10.1186/s12911-023-02313-1)
Supplement: Supplementary file 1 — Additional file 1: Supplementary file A. PRISMA 2020 checklist. Supplementary file B. Summary of included studies for systematic review. Supplementary file C. Studies included in meta-analysis. Supplementary file D. Forest plots [file 12911_2023_2313_MOESM1_ESM.docx]

# Supplementary file A. PRISMA 2020 checklist

| **Section and Topic** | **Item #** | **Checklist item** | **Location where item is reported** |
| --- | --- | --- | --- |
| **TITLE** | | |  |
| Title | 1 | Identify the report as a systematic review. | P. 1, Title |
| **ABSTRACT** | | |  |
| Abstract | 2 | See the PRISMA 2020 for Abstracts checklist. | P. 1 |
| **INTRODUCTION** | | |  |
| Rationale | 3 | Describe the rationale for the review in the context of existing knowledge. | PP. 2 – 3 |
| Objectives | 4 | Provide an explicit statement of the objective(s) or question(s) the review addresses. | P. 3 |
| **METHODS** | | |  |
| Eligibility criteria | 5 | Specify the inclusion and exclusion criteria for the review and how studies were grouped for the syntheses. | P. 4, Eligibility criteria and study selection |
| Information sources | 6 | Specify all databases, registers, websites, organisations, reference lists and other sources searched or consulted to identify studies. Specify the date when each source was last searched or consulted. | P. 3, Data sources and search strategy |
| Search strategy | 7 | Present the full search strategies for all databases, registers and websites, including any filters and limits used. | P. 4, Table 1 |
| Selection process | 8 | Specify the methods used to decide whether a study met the inclusion criteria of the review, including how many reviewers screened each record and each report retrieved, whether they worked independently, and if applicable, details of automation tools used in the process. | P. 4, Eligibility criteria and study selection |
| Data collection process | 9 | Specify the methods used to collect data from reports, including how many reviewers collected data from each report, whether they worked independently, any processes for obtaining or confirming data from study investigators, and if applicable, details of automation tools used in the process. | P. 4, Eligibility criteria and study selection |
| Data items | 10a | List and define all outcomes for which data were sought. Specify whether all results that were compatible with each outcome domain in each study were sought (e.g. for all measures, time points, analyses), and if not, the methods used to decide which results to collect. | P. 5, Data extraction |
|  | 10b | List and define all other variables for which data were sought (e.g. participant and intervention characteristics, funding sources). Describe any assumptions made about any missing or unclear information. | P. 5, Data extraction |
| Study risk of bias assessment | 11 | Specify the methods used to assess risk of bias in the included studies, including details of the tool(s) used, how many reviewers assessed each study and whether they worked independently, and if applicable, details of automation tools used in the process. | P. 5, Study quality assessment |
| Effect measures | 12 | Specify for each outcome the effect measure(s) (e.g. risk ratio, mean difference) used in the synthesis or presentation of results. | P. 6, Data analytic procedures |
| Synthesis methods | 13a | Describe the processes used to decide which studies were eligible for each synthesis (e.g. tabulating the study intervention characteristics and comparing against the planned groups for each synthesis (item #5)). | NA |
|  | 13b | Describe any methods required to prepare the data for presentation or synthesis, such as handling of missing summary statistics, or data conversions. | NA |
|  | 13c | Describe any methods used to tabulate or visually display results of individual studies and syntheses. | NA |
|  | 13d | Describe any methods used to synthesize results and provide a rationale for the choice(s). If meta-analysis was performed, describe the model(s), method(s) to identify the presence and extent of statistical heterogeneity, and software package(s) used. | P. 13, Meta-analysis results |
|  | 13e | Describe any methods used to explore possible causes of heterogeneity among study results (e.g. subgroup analysis, meta-regression). | P. 17, Moderator analysis results |
|  | 13f | Describe any sensitivity analyses conducted to assess robustness of the synthesized results. | NA |
| Reporting bias assessment | 14 | Describe any methods used to assess risk of bias due to missing results in a synthesis (arising from reporting biases). | NA |
| Certainty assessment | 15 | Describe any methods used to assess certainty (or confidence) in the body of evidence for an outcome. | NA |
| **RESULTS** | | |  |
| Study selection | 16a | Describe the results of the search and selection process, from the number of records identified in the search to the number of studies included in the review, ideally using a flow diagram. | PP. 4 - 5, Eligibility criteria and study selection, Fig. 1 |
|  | 16b | Cite studies that might appear to meet the inclusion criteria, but which were excluded, and explain why they were excluded. | P. 5, Fig. 1 |
| Study characteristics | 17 | Cite each included study and present its characteristics. | P. 32 - 69, Appendix B. |
| Risk of bias in studies | 18 | Present assessments of risk of bias for each included study. | P. 5, Study quality assessment |
| Results of individual studies | 19 | For all outcomes, present, for each study: (a) summary statistics for each group (where appropriate) and (b) an effect estimate and its precision (e.g. confidence/credible interval), ideally using structured tables or plots. | P. 32 - 69, Appendix B |
| Results of syntheses | 20a | For each synthesis, briefly summarise the characteristics and risk of bias among contributing studies. | P. 5, Study quality assessment,  P. 7, Characteristics of eligible studies |
|  | 20b | Present results of all statistical syntheses conducted. If meta-analysis was done, present for each the summary estimate and its precision (e.g. confidence/credible interval) and measures of statistical heterogeneity. If comparing groups, describe the direction of the effect. | . 6, Results, P. 17, Meta-analysis results |
|  | 20c | Present results of all investigations of possible causes of heterogeneity among study results. | P. 16, Table 5 |
|  | 20d | Present results of all sensitivity analyses conducted to assess the robustness of the synthesized results. | NA |
| Reporting biases | 21 | Present assessments of risk of bias due to missing results (arising from reporting biases) for each synthesis assessed. | NA |
| Certainty of evidence | 22 | Present assessments of certainty (or confidence) in the body of evidence for each outcome assessed. | P. 16, Table 5 |
| **DISCUSSION** | | |  |
| Discussion | 23a | Provide a general interpretation of the results in the context of other evidence. | P. 22, Discussion |
|  | 23b | Discuss any limitations of the evidence included in the review. | P. 27, Limitations |
|  | 23c | Discuss any limitations of the review processes used. | P. 27, Limitations |
|  | 23d | Discuss implications of the results for practice, policy, and future research. | P. 26 - 27, Theoretical implications, Practical implications |
| **OTHER INFORMATION** | | |  |
| Registration and protocol | 24a | Provide registration information for the review, including register name and registration number, or state that the review was not registered. | NA |
|  | 24b | Indicate where the review protocol can be accessed, or state that a protocol was not prepared. | NA |
|  | 24c | Describe and explain any amendments to information provided at registration or in the protocol. | NA |
| Support | 25 | Describe sources of financial or non-financial support for the review, and the role of the funders or sponsors in the review. | P. 28, Funding |
| Competing interests | 26 | Declare any competing interests of review authors. | P. 28, Conflicts of interest |
| Availability of data, code and other materials | 27 | Report which of the following are publicly available and where they can be found: template data collection forms; data extracted from included studies; data used for all analyses; analytic code; any other materials used in the review. | P. 28, Availability of data and materials |

# Supplementary file B. Summary of included studies for systematic review

| Study | Theory | Approaches | Area | n | Antecedents | DCT acceptance | Findings |
| --- | --- | --- | --- | --- | --- | --- | --- |
| Abramova et al. [3] | Privacy calculus theory | Qualitative+Quantitative | Europe | 589 | Social risks | Actual use of DCT | **(-) |
|  |  |  |  |  | Privacy risks | Actual use of DCT | n.s. |
|  |  |  |  |  | Individual benefits | Intention to use DCT | *** |
|  |  |  |  |  | Social risks | Intention to use DCT | ***(-) |
|  |  |  |  |  | Social benefits | Intention to use DCT | *** |
|  |  |  |  |  | Privacy risks | Intention to use DCT | *(-) |
| Ahmad et al. [43] | Theory of planned behavior, Theory of reasoned action | Quantitative | Asia | 302 | Attitude towards epidemic prevention | Intention to adopt epidemic prevention | * |
|  |  |  |  |  | Risk aversion | Intention to adopt epidemic prevention | * |
|  |  |  |  |  | Government's guidelines on epidemic prevention | Intention to adopt epidemic prevention | * |
|  |  |  |  |  | Epidemic knowledge | Intention to adopt epidemic prevention | * |
|  |  |  |  |  | Moral norms | Intention to adopt epidemic prevention | n.s. |
|  |  |  |  |  | Subjective norms | Intention to adopt epidemic prevention | * |
|  |  |  |  |  | Perceived feasibility to adopt epidemic prevention | Intention to adopt epidemic prevention | *(-) |
|  |  |  |  |  | Risk perception | Intention to adopt epidemic prevention | * |
|  |  |  |  |  | Perceived behavioral control | Intention to adopt epidemic prevention | * |
| Akinnuwesi et al. [44] | Unified theory of acceptance and use of technology | Quantitative | Africa | 650 | Facilitating condition | Intention to adopt epidemic prevention | ** |
|  |  |  |  |  | Government expectancy and benefit | Intention to adopt epidemic prevention | n.s. |
|  |  |  |  |  | Social influence | Intention to adopt epidemic prevention | ** |
|  |  |  |  |  | Organization influence and benefit | Intention to adopt epidemic prevention | n.s. |
|  |  |  |  |  | Perceived cost | Intention to adopt epidemic prevention | n.s. |
|  |  |  |  |  | Effort expectancy | Intention to adopt epidemic prevention | n.s. |
|  |  |  |  |  | Performance expectancy | Intention to adopt epidemic prevention | ** |
|  |  |  |  |  | Public awareness | Intention to adopt epidemic prevention | n.s. |
|  |  |  |  |  | Data security and privacy | Intention to adopt epidemic prevention | n.s. |
| Ali and Dang [45] | Trust theory, Protection motivation theory | Quantitative | Oceania | 261 | Fear of COVID-19 | NZ COVID Tracer app usage behavior | n.s. |
|  |  |  |  |  | Knowing someone infected with COVID-19 | NZ COVID Tracer app usage behavior | n.s. |
|  |  |  |  |  | Self-perceived health | NZ COVID Tracer app usage behavior | n.s. |
|  |  |  |  |  | Trust in data privacy protection | NZ COVID Tracer app usage behavior | *** |
|  |  |  |  |  | Trust in social distancing practice | NZ COVID Tracer app usage behavior | n.s. |
| Alkhalifah and Bukar [29] | Task-technology fit, Technology acceptance model, Protection motivation theory | Quantitative | Africa | 309 | Perceived ease of use | Tawakkalna BI use | *** |
|  |  |  |  |  | Perceived usefulness | Tawakkalna BI use | *** |
|  |  |  |  |  | Privacy risk | Tawakkalna BI use | n.s. |
|  |  |  |  |  | Social interaction | Tawakkalna BI use | n.s. |
|  |  |  |  |  | Social risks | Tawakkalna BI use | n.s. |
|  |  |  |  |  | Task-technology fit | Tawakkalna BI use | *** |
| Alsaad and Al-Okaily [25] | Protection motivation theory | Quantitative | Asia | 307 | Protection motivation | Intention to use exposure detection apps | *** |
| Alshami et al. [30] | Unified theory of acceptance and use of technology | Quantitative | Asia | 150 | App-related privacy concern | Adoption of MySejahtera | n.s. |
|  |  |  |  |  | Performance expectancy | Adoption of MySejahtera | * |
|  |  |  |  |  | facilitating condition | Adoption of MySejahtera | *** |
|  |  |  |  |  | effort expectancy | Adoption of MySejahtera | * |
|  |  |  |  |  | social influence | Adoption of MySejahtera | * |
| Behne et al. [11] | Technology acceptance model | Quantitative | Europe | 1971 | Perceived anxiety of infecting others | Intention to use DCT | ** |
|  |  |  |  |  | Perceived anxiety of infection | Intention to use DCT | n.s. |
|  |  |  |  |  | Subjective norm and reputation | Intention to use DCT | n.s. |
|  |  |  |  |  | Perceived ease of use | Intention to use DCT | * |
|  |  |  |  |  | Perceived usefulness | Intention to use DCT | *** |
|  |  |  |  |  | Altruism | Intention to use DCT | ** |
|  |  |  |  |  | Personal innovativeness | Intention to use DCT | ** |
|  |  |  |  |  | Privacy concerns | Intention to use DCT | ***(-) |
|  |  |  |  |  | Trust in technology | Intention to use DCT | *** |
| Bradshaw et al. [31] | Self-determination theory | Quantitative | Americas | 888 | Perceived government legitimacy | Intention to download DCT | *** |
|  |  |  |  |  | Message framing | Intention to download DCT | ** |
|  |  |  |  |  | Information safety | Intention to download DCT | * |
|  |  |  | Oceania | 1117 | Message framing | Intention to download DCT | n.s. |
|  |  |  |  |  | Information safety | Intention to download DCT | ** |
| Chan and Saqib [32] | Social conservatism | Quantitative | Oceania | 202 | COVID-19 concerns | Choice to download DCT | *(-) |
|  |  |  |  |  | Social conservatism | Choice to download DCT | * |
|  |  |  | Europe | 471 | COVID-19 concerns | Intention to download DCT | **(-) |
|  |  |  | Americas | 1005 | COVID-19 concerns | Willing to download DCT | **(-) |
|  |  |  |  |  | State political ideology | Willing to download DCT | ** |
| Chan et al. [33] | Unified theory of acceptance and use of technology 2 | Quantitative | Asia | 401 | Facilitating condition | Intention to use MySejahtera application | *** |
|  |  |  |  |  | Habit | Intention to use MySejahtera application | *** |
|  |  |  |  |  | Social influence | Intention to use MySejahtera application | * |
|  |  |  |  |  | Effort expectancy | Intention to use MySejahtera application | n.s. |
|  |  |  |  |  | Performance expectancy | Intention to use MySejahtera application | n.s. |
|  |  |  |  |  | Perceived privacy credibility | Intention to use MySejahtera application | ** |
| Chen et al. [26] | Social exchange theory | Quantitative | Americas | 365 | Affective trust | Willingness to disclose | *** |
|  |  |  |  |  | Cognitive trust | Willingness to disclose | *** |
|  |  |  |  |  | Affective trust | Willingness to falsify | *** |
|  |  |  |  |  | Cognitive trust | Willingness to falsify | p<0.1 |
| Chopdar [46] | Unified theory of acceptance and use of technology, Health belief model | Quantitative | Asia | 307 | Facilitating condition | Behavioral intention | ** |
|  |  |  |  |  | Social influence | Behavioral intention | ** |
|  |  |  |  |  | Effort expectancy | Behavioral intention | n.s. |
|  |  |  |  |  | Performance expectancy | Behavioral intention | *** |
|  |  |  |  |  | Perceived privacy risk | Behavioral intention | ***(-) |
|  |  |  |  |  | Perceived security risk | Behavioral intention | ***(-) |
| Chuenyindee et al. [47] | Technology acceptance model 2, Protection motivation theory | Quantitative | Asia | 800 | Attitude toward using CTA | Intention to use DCT | ** |
| Cocosila et al. [48] | Theory of consumption values | Quantitative | Americas | 309 | Critical mass | Behavioral intention | *** |
|  |  |  |  |  | Health information seeking | Behavioral intention | ** |
|  |  |  |  |  | Social influence | Behavioral intention | n.s. |
|  |  |  |  |  | Perceived value | Behavioral intention | *** |
|  |  |  |  |  | Personal IT innovation | Behavioral intention | n.s. |
| Dowthwaite et al. [49] | Trust theory, Technology acceptance model 2 | Quantitative | Europe | 1001 | Perceived ease of use | Intention to use | ** |
|  |  |  |  |  | Perceived usefulness | Intention to use | ** |
|  |  |  |  |  | Trust | Intention to use | *** |
| Duan and Deng [34] | Unified theory of acceptance and use of technology, Privacy calculus theory | Quantitative | Oceania | 307 | Facilitating condition | Adoption intention | n.s. |
|  |  |  |  |  | Social influence | Adoption intention | ** |
|  |  |  |  |  | Effort expectancy | Adoption intention | *** |
|  |  |  |  |  | Performance expectancy | Adoption intention | n.s. |
|  |  |  |  |  | Perceived value of information disclosure | Adoption intention | *** |
| Duan and Deng [35] | Privacy calculus theory, APCO model | Quantitative | Oceania | 307 | Perceived benefit | Adoption intention | *** |
|  |  |  |  |  | P:erceived privacy risk | Adoption intention | *(-) |
|  |  |  |  |  | Trust | Adoption intention | *** |
| Dzandu [50] | Technology acceptance model | Quantitative | Europe | 2512 | Perception of gov | Voluntary acceptance | *** |
|  |  |  |  |  | Trust in gov | Voluntary acceptance | ***(-) |
| Farrell et al. [51] | Technology acceptance model | Quantitative | Europe | 407 | Subjective norm | Behavioral intention | ** |
|  |  |  |  |  | Perceived ease of use | Behavioral intention | n.s. |
|  |  |  |  |  | Perceived usefulness | Behavioral intention | ** |
|  |  |  |  |  | Information privacy concern | Behavioral intention | **(-) |
|  |  |  |  |  | Trust | Behavioral intention | ** |
| Fernandes and Costa [52] | Privacy calculus theory | Quantitative | Europe | 504 | Lack of control | Willingness to disclose personal information | n.s. |
|  |  |  |  |  | Personal benefits | Willingness to disclose personal information | *** |
|  |  |  |  |  | Societal benefits | Willingness to disclose personal information | *** |
|  |  |  |  |  | Privacy concern | Willingness to disclose personal information | *(-) |
| Fox et al. [13] | Privacy calculus theory, Social exchange theory | Quantitative | Europe | 405 | Social influence | Adoption intention | *** |
|  |  |  |  |  | Perceived health benefits | Adoption intention | ** |
|  |  |  |  |  | Reciprocal benefits | Adoption intention | *** |
|  |  |  |  |  | Privacy concerns | Adoption intention | n.s. |
| Fox et al. [53] | APCO model, Privacy calculus theory, Social contract theory | Quantitative | Americas | 1175 | Social influence | Intention to continue use | n.s. |
|  |  |  |  |  | Perceived health benefits | Intention to continue use | n.s. |
|  |  |  |  |  | Reciprocal benefits | Intention to continue use | *** |
|  |  |  |  |  | Perceived privacy of the APP | Intention to continue use | ** |
|  |  |  |  |  | Reciprocal benefits | Willingness to disclose data | ** |
|  |  |  |  |  | Perceived privacy of the APP | Willingness to disclose data | ** |
| Geber and Friemel [14] | Technology acceptance model, Protection motivation theory, Social norm | Quantitative | Europe | 1076 | Descriptive norm personal environment | Intention to adopt DCT | n.s. |
|  |  |  |  |  | Descriptive norm population | Intention to adopt DCT | * |
|  |  |  |  |  | Injunctive norm personal environment | Intention to adopt DCT | ** |
|  |  |  |  |  | Injunctive norm population | Intention to adopt DCT | n.s. |
|  |  |  |  |  | Perceived ease of use | Intention to adopt DCT | *** |
|  |  |  |  |  | Perceived usefulness | Intention to adopt DCT | *** |
|  |  |  |  |  | Susceptibility to data misuse | Intention to adopt DCT | n.s. |
|  |  |  |  |  | Severity to data misuse | Intention to adopt DCT | **(-) |
|  |  |  |  |  | Severity of COVID-19 | Intention to adopt DCT | * |
|  |  |  |  |  | Susceptibility to COVID-19 | Intention to adopt DCT | *(-) |
| Goyal et al. [15] | Theory of consumption values, Privacy theory | Quantitative | Asia | 850 | Functional value | Intention to use | * |
|  |  |  |  |  | Epistemic value | Intention to use | n.s. |
|  |  |  |  |  | Conditional value | Intention to use | * |
|  |  |  |  |  | Convenience value | Intention to use | * |
|  |  |  |  |  | Privacy concern | Intention to use | n.s. |
| Guazzini et al. [54] | Theory of reasoned action, Technology acceptance model, Unified theory of acceptance and use of technology | Quantitative | Europe | 501 | Attitude toward DCT | Intention to adopt | ** |
|  |  |  |  |  | Social influence | Intention to adopt | ** |
|  |  |  |  |  | Perceived severity of COVID-19 | Intention to adopt | p=0.05 |
|  |  |  |  |  | Likelihood of infection | Intention to adopt | p=0.05 |
| Guillon [55] | Health belief model | Quantitative | Europe | 1042 | Endorsement of COVID-19 conspiracy theories | Use of DCT | ** |
|  |  |  |  |  | Perceived efficacy of DCT | Use of DCT | *** |
|  |  |  |  |  | Perceived guarantee on data privacy | Use of DCT | *** |
|  |  |  |  |  | Perceived health impacts of the COVID-19 epidemics in France | Use of DCT | n.s. |
|  |  |  |  |  | Trust in the government | Use of DCT | n.s. |
| Guo et al. [16] | Technology acceptance model, Unified theory of acceptance and use of technology, Theory of planned behavior | Quantitative+Qualitative | Asia | 260 | Perceived ease of use of RLTS tag | Willingness to use DCT | * |
|  |  |  |  |  | Acceptance of the use of RLTS tag | Willingness to use DCT | *** |
|  |  |  |  |  | Privacy concerns on the use of RLTS | Willingness to use DCT | n.s. |
| Harborth and Pape [56] | APCO model | Quantitative | Europe | 1660 | Perceived benefits of DCT | Use of DCT | *** |
|  |  |  |  |  | Privacy concerns regarding DCT | Use of DCT | ***(-) |
|  |  |  |  |  | Trust in German healthcare system | Use of DCT | *(-) |
| Harborth et al. [57] | Health belief model | Quantitative | Europe | 1571 | Intrinsic motivation | Use of DCT | *** |
|  |  |  |  |  | Extrinsic motivation | Use of DCT | *** |
|  |  |  |  |  | Perceived benefits | Use of DCT | * |
|  |  |  |  |  | Perceived technical barriers | Use of DCT | ***(-) |
|  |  |  |  |  | Privacy concerns | Use of DCT | ***(-) |
|  |  |  |  |  | Perceived susceptibility | Use of DCT | * |
|  |  |  |  |  | Perceived medical sequences | Use of DCT | n.s. |
|  |  |  |  |  | Perceived severity on others | Use of DCT | ***(-) |
|  |  |  |  |  | Perceived social consequences | Use of DCT | n.s. |
| Hassandoust et al. [58] | Privacy calculus theory | Quantitative | Americas | 856 | Social influence | Intention to install DCT | *** |
|  |  |  |  |  | Perceived effort | Intention to install DCT | *(-) |
|  |  |  |  |  | Contact tracing benefits | Intention to install DCT | *** |
|  |  |  |  |  | Personal innovativeness | Intention to install DCT | *** |
|  |  |  |  |  | Risk beliefs | Intention to install DCT | ***(-) |
|  |  |  |  |  | Trusting beliefs | Intention to install DCT | n.s. |
|  |  |  |  |  | Voluntariness | Intention to install DCT | *(-) |
| Hauff and Nilsson [59] | Privacy calculus theory | Quantitative | Europe | 1007 | Attitude towards technology | Willingness to use DCT | *** |
|  |  |  |  |  | Hedonic usefulness | Willingness to use DCT | * |
|  |  |  |  |  | Utilitarian usefulness | Willingness to use DCT | n.s. |
|  |  |  |  |  | Pro-social usefulness | Willingness to use DCT | *** |
|  |  |  |  |  | Privacy concern | Willingness to use DCT | ***(-) |
| Horvath et al. [60] | Technology acceptance model | Quantitative | Europe | 1026 | Compliance | Continued use | n.s. |
|  |  |  |  |  | Mobility | Continued use | n.s. |
|  |  |  |  |  | Social norms | Continued use | n.s. |
|  |  |  |  |  | Ease of use | Continued use | n.s. |
|  |  |  |  |  | Perceived usefulness | Continued use | n.s. |
|  |  |  |  |  | Privacy concern | Continued use | n.s. |
|  |  |  |  |  | Transparent evidence | Continued use | n.s. |
|  |  |  |  |  | More information on data usage | Continued use | n.s. |
|  |  |  |  |  | Evidence on vulnerable groups | Continued use | n.s. |
|  |  |  |  |  | Trust in UK Govt | Continued use | n.s. |
|  |  |  |  | 2500 | Compliance | Initial adoption | * |
|  |  |  |  |  | Mobility | Initial adoption | n.s. |
|  |  |  |  |  | Social norms | Initial adoption | *** |
|  |  |  |  |  | Ease of use | Initial adoption | *** |
|  |  |  |  |  | Perceived usefulness | Initial adoption | n.s. |
|  |  |  |  |  | Privacy concern | Initial adoption | *** |
|  |  |  |  |  | Transparent evidence | Initial adoption | * |
|  |  |  |  |  | More information on data usage | Initial adoption | n.s. |
|  |  |  |  |  | Evidence on vulnerable groups | Initial adoption | n.s. |
|  |  |  |  |  | Trust in UK Govt | Initial adoption | n.s. |
|  |  |  |  | 1474 | Compliance | New adoption | n.s. |
|  |  |  |  |  | Mobility | New adoption | n.s. |
|  |  |  |  |  | Social norms | New adoption | * |
|  |  |  |  |  | Ease of use | New adoption | n.s. |
|  |  |  |  |  | Perceived usefulness | New adoption | n.s. |
|  |  |  |  |  | Privacy concern | New adoption | * |
|  |  |  |  |  | Transparent evidence | New adoption | n.s. |
|  |  |  |  |  | More information on data usage | New adoption | n.s. |
|  |  |  |  |  | Evidence on vulnerable groups | New adoption | n.s. |
|  |  |  |  |  | Trust in UK Govt | New adoption | * |
| Huang et al. [36] | Technology acceptance model, Health information technology acceptance model | Quantitative | Asia | 857 | Perceived DCT as useful and necessary | DCT acceptance | *** |
|  |  |  |  |  | Concerns about personal data collected by DCT | DCT acceptance | *** |
|  |  |  |  | 1274 | Liberal individualism | DCT acceptance | n.s. |
|  |  |  |  |  | Perceived DCT as useful and necessary | DCT acceptance | *** |
|  |  |  |  |  | Concerns about personal data collected by DCT | DCT acceptance | *** |
|  |  |  |  | 1812 | Liberal individualism | DCT acceptance | n.s. |
|  |  |  |  |  | Perceived DCT as useful and necessary | DCT acceptance | *** |
|  |  |  |  |  | Values personal and loved ones' health | DCT acceptance | * |
|  |  |  |  |  | Concerns about personal data collected by DCT | DCT acceptance | *** |
|  |  |  |  | 857 | Liberal individualism | DCT adoption | n.s. |
|  |  |  |  |  | Perceived DCT as useful and necessary | DCT adoption | * |
|  |  |  |  |  | Concerns about personal data collected by DCT | DCT adoption | ** |
|  |  |  |  | 1274 | Ease of use | DCT adoption | *** |
|  |  |  |  |  | Perceived DCT as useful and necessary | DCT adoption | * |
|  |  |  |  |  | Concerns about personal data collected by DCT | DCT adoption | *** |
|  |  |  |  | 1812 | Liberal individualism | DCT adoption | * |
|  |  |  |  |  | Ease of use | DCT adoption | *** |
|  |  |  |  |  | Perceived DCT as useful and necessary | DCT adoption | *** |
|  |  |  |  |  | Concerns about personal data collected by DCT | DCT adoption | *** |
| Jahari et al. [61] | Technology acceptance model 2 | Quantitative | Americas | 269 | Reputation | Intention to use | *** |
|  |  |  |  |  | Reciprocity | Intention to use | *** |
|  |  |  |  |  | Privacy concerns | Intention to use | *** |
|  |  |  |  |  | Trust | Intention to use | n.s. |
| Jansen-Kosterink et al. [37] | Health belief model, Health belief model | Quantitative | Europe | 238 | Attitude toward technology | Intention to use | * |
|  |  |  |  |  | Fear of COVID-19 | Intention to use | *** |
|  |  |  |  |  | Perceived health | Intention to use | n.s. |
| Jörling et al. [38] | Technology acceptance model 2 | Quantitative | Europe | 957 | Fear of COVID-19 | Willingness to disclose | *** |
|  |  |  |  |  | Subjective norm | Willingness to disclose | *** |
|  |  |  |  |  | Perceived ease of use | Willingness to disclose | *** |
|  |  |  |  |  | Perceived prosocial benefits | Willingness to disclose | *** |
|  |  |  |  |  | Trust in public institution | Willingness to disclose | *** |
| Kaspar [62] | Protection motivation theory | Quantitative | Europe | 406 | Vulnerability of data misuse | Motivation for using a contact tracing app | *** |
|  |  |  |  |  | Severity of data misuse | Motivation for using a contact tracing app | * |
|  |  |  |  |  | General trust in official app providers | Motivation for using a contact tracing app | *** |
|  |  |  |  |  | Trust in other people's social distancing behavior | Motivation for using a contact tracing app | * |
| Kostka and Habich-Sobiegalla [63] | Technology acceptance model, Unified theory of acceptance and use of technology, Privacy calculus theory | Quantitative | Europe | 2083 | Capability-central government | CTA acceptance | * |
|  |  |  |  |  | Capability-experts | CTA acceptance | n.s. |
|  |  |  |  |  | Capability-local government | CTA acceptance | * |
|  |  |  |  |  | Capability-private enterprises | CTA acceptance | * |
|  |  |  |  |  | Conspiracy | CTA acceptance | n.s. |
|  |  |  |  |  | Similar app usage | CTA acceptance | ** |
|  |  |  |  |  | CTA experience | CTA acceptance | *** |
|  |  |  |  |  | Financial situation-worse | CTA acceptance | *(-) |
|  |  |  |  |  | Understanding | CTA acceptance | n.s. |
|  |  |  |  |  | Health concern-myself | CTA acceptance | * |
|  |  |  |  |  | Health concern-family | CTA acceptance | * |
|  |  |  |  |  | Health concern-friends | CTA acceptance | n.s. |
|  |  |  |  |  | Health concern-none | CTA acceptance | n.s. |
|  |  |  |  |  | Perceived consequences-fewer infection | CTA acceptance | *** |
|  |  |  |  |  | Perceived consequences-better information | CTA acceptance | *** |
|  |  |  |  |  | CTA effectiveness | CTA acceptance | *** |
|  |  |  |  |  | Perceived consequences-surveillance | CTA acceptance | ***(-) |
|  |  |  |  |  | Perceived consequences-privacy violation | CTA acceptance | ***(-) |
|  |  |  |  |  | Second wave | CTA acceptance | ** |
|  |  |  |  |  | Capability-individual | CTA acceptance | n.s. |
|  |  |  |  |  | Trust in the government-not at all | CTA acceptance | **(-) |
|  |  |  |  |  | Trust in the government-not much | CTA acceptance | *** |
|  |  |  |  |  | Trust in the government-somewhat | CTA acceptance | *** |
|  |  |  |  |  | Trust in the government-a lot | CTA acceptance | *** |
|  |  |  | Americas | 2180 | Capability-central government | CTA acceptance | n.s. |
|  |  |  |  |  | Capability-experts | CTA acceptance | ** |
|  |  |  |  |  | Capability-local government | CTA acceptance | n.s. |
|  |  |  |  |  | Capability-private enterprises | CTA acceptance | n.s. |
|  |  |  |  |  | Conspiracy | CTA acceptance | n.s. |
|  |  |  |  |  | Similar app usage | CTA acceptance | *** |
|  |  |  |  |  | CTA experience | CTA acceptance | ** |
|  |  |  |  |  | Financial situation-worse | CTA acceptance | n.s. |
|  |  |  |  |  | Understanding | CTA acceptance | n.s. |
|  |  |  |  |  | Health concern-myself | CTA acceptance | ** |
|  |  |  |  |  | Health concern-family | CTA acceptance | n.s. |
|  |  |  |  |  | Health concern-friends | CTA acceptance | n.s. |
|  |  |  |  |  | Health concern-none | CTA acceptance | n.s. |
|  |  |  |  |  | Perceived consequences-fewer infection | CTA acceptance | *** |
|  |  |  |  |  | Perceived consequences-better information | CTA acceptance | *** |
|  |  |  |  |  | CTA effectiveness | CTA acceptance | *** |
|  |  |  |  |  | Perceived consequences-surveillance | CTA acceptance | ***(-) |
|  |  |  |  |  | Perceived consequences-privacy violation | CTA acceptance | ***(-) |
|  |  |  |  |  | Second wave | CTA acceptance | *** |
|  |  |  |  |  | Capability-individual | CTA acceptance | n.s. |
|  |  |  |  |  | Trust in the government-not at all | CTA acceptance | ***(-) |
|  |  |  |  |  | Trust in the government-not much | CTA acceptance | n.s. |
|  |  |  |  |  | Trust in the government-somewhat | CTA acceptance | ** |
|  |  |  |  |  | Trust in the government-a lot | CTA acceptance | *** |
|  |  |  | Asia | 2201 | Capability-central government | CTA acceptance | ** |
|  |  |  |  |  | Capability-experts | CTA acceptance | ** |
|  |  |  |  |  | Capability-local government | CTA acceptance | n.s. |
|  |  |  |  |  | Capability-private enterprises | CTA acceptance | * |
|  |  |  |  |  | Conspiracy | CTA acceptance | n.s. |
|  |  |  |  |  | Similar app usage | CTA acceptance | n.s. |
|  |  |  |  |  | CTA experience | CTA acceptance | *** |
|  |  |  |  |  | Financial situation-worse | CTA acceptance | n.s. |
|  |  |  |  |  | Understanding | CTA acceptance | *** |
|  |  |  |  |  | Health concern-myself | CTA acceptance | * |
|  |  |  |  |  | Health concern-family | CTA acceptance | n.s. |
|  |  |  |  |  | Health concern-friends | CTA acceptance | n.s. |
|  |  |  |  |  | Health concern-none | CTA acceptance | n.s. |
|  |  |  |  |  | Perceived consequences-fewer infection | CTA acceptance | n.s. |
|  |  |  |  |  | Perceived consequences-better information | CTA acceptance | *** |
|  |  |  |  |  | CTA effectiveness | CTA acceptance | *** |
|  |  |  |  |  | Perceived consequences-surveillance | CTA acceptance | n.s. |
|  |  |  |  |  | Perceived consequences-privacy violation | CTA acceptance | **(-) |
|  |  |  |  |  | Second wave | CTA acceptance | ** |
|  |  |  |  |  | Capability-individual | CTA acceptance | n.s. |
|  |  |  |  |  | Trust in the government-not at all | CTA acceptance | n.s. |
|  |  |  |  |  | Trust in the government-not much | CTA acceptance | n.s. |
|  |  |  |  |  | Trust in the government-somewhat | CTA acceptance | *** |
|  |  |  |  |  | Trust in the government-a lot | CTA acceptance | *** |
| Krüger et al. [64] | Technology acceptance model | Quantitative | Europe | 2682 | Perceived anxiety of infection | Behavioral intention to use DCT | n.s. |
|  |  |  |  |  | Perceived anxiety of infecting others | Behavioral intention to use DCT | *** |
|  |  |  |  |  | Reputation | Behavioral intention to use DCT | n.s. |
|  |  |  |  |  | Perceived usefulness | Behavioral intention to use DCT | *** |
|  |  |  |  |  | Personal innovativeness | Behavioral intention to use DCT | *** |
|  |  |  |  |  | Altruism | Behavioral intention to use DCT | *** |
|  |  |  |  |  | Privacy concerns | Behavioral intention to use DCT | ***(-) |
|  |  |  |  |  | Trust in technology | Behavioral intention to use DCT | *** |
| Kulyk et al. [65] | Privacy theory | Quantitative | Europe | 284 | Privacy concerns | Adoption of DCT | *** |
|  |  |  |  |  | Trust | Adoption of DCT | *** |
| Kurtaliqi et al. [66] | Theory of valuation | Quantitative | Europe | 820 | Perceived value of StopCovid app | Word of mouth | ***(-) |
|  |  |  |  |  | Trust in government | Word of mouth | n.s. |
|  |  |  |  |  | Trust in the future | Word of mouth | * |
|  |  |  |  |  | Wellbeing | Word of mouth | *** |
| Lee et al. [67] | Theory of normative social behavior | Quantitative | Asia | 1137 | COVID-19-related worry | DCT use intention | n.s. |
|  |  |  |  |  | Knowledge about contact tracing | DCT use intention | *** |
|  |  |  |  |  | Descriptive norms | DCT use intention | *** |
|  |  |  |  |  | Injunctive norms | DCT use intention | *** |
|  |  |  |  |  | Perceived community | DCT use intention | ** |
|  |  |  |  |  | Health status | DCT use intention | n.s. |
| Lin et al. [68] | Diffusion innovation theory, Internet users' information privacy concern | Quantitative | Oceania | 209 | Compatibility | Intention to use | *** |
|  |  |  |  |  | Perceived ease of use | Intention to use | n.s. |
|  |  |  |  |  | Risk belief | Intention to use | n.s. |
|  |  |  |  |  | Relative advantage | Intention to use | *** |
|  |  |  |  |  | Trusting belief | Intention to use | * |
| Meier et al. [17] | Privacy calculus theory | Quantitative | Europe | 952 | Factual knowledge | App usage intention | n.s. |
|  |  |  |  |  | Perceived benefits | App usage intention | *** |
|  |  |  |  |  | Privacy concerns | App usage intention | n.s. |
|  |  |  |  |  | Trust | App usage intention | n.s. |
| Nguyen and Nguyen [39] | Unified theory of acceptance and use of technology | Quantitative | Asia | 224 | Facilitating conditions | Behavioral intention to use DCT | n.s. |
|  |  |  |  |  | Social influence | Behavioral intention to use DCT | n.s. |
|  |  |  |  |  | Effort expectancy | Behavioral intention to use DCT | n.s. |
|  |  |  |  |  | Performance expectancy | Behavioral intention to use DCT | * |
|  |  |  |  |  | Privacy risk | Behavioral intention to use DCT | *(-) |
|  |  |  |  |  | Trust | Behavioral intention to use DCT | * |
| Nguyen et al. [69] | Technology acceptance model | Quantitative | Americas | 288 | Health information orientation to COVID-19 | Adoption intention | * |
|  |  |  |  |  | Perceived ease of use | Adoption intention | n.s. |
|  |  |  |  |  | Health risk perception from COVID-19 | Adoption intention | ** |
|  |  |  |  |  | Health consciousness | Adoption intention | n.s. |
|  |  |  |  |  | Perceived usefulness | Adoption intention | ** |
| Nguyen et al. [70] | Health belief model | Quantitative | Asia | 219 | Perceived benefits | Use of DCT | ** |
|  |  |  |  |  | Privacy concerns | Use of DCT | **(-) |
| Ntsiful et al. [71] | Health belief model, Theory of reasoned action | Quantitative | Africa | 137 | Attitude | Intention to adopt DCT | *** |
| Nunes et al. [72] | Unified theory of acceptance and use of technology | Quantitative | Europe | 9555 | Facilitating conditions | Intention to use | *** |
|  |  |  |  |  | Performance expectancy | Intention to use | *** |
|  |  |  |  |  | Security | Intention to use | *** |
|  |  |  |  |  | Safety measures | Intention to use | *** |
| Oldeweme et al. [73] | Uncertainty reduction theory | Quantitative | Europe | 1003 | Social influence | Intention to use | *** |
|  |  |  |  |  | Initial trust | Intention to use | *** |
| Oyibo and Morita [74] | Unified theory of acceptance and use of technology | Quantitative | Americas | 196 | Perceived compatibility | Intention to use | * |
|  |  |  |  |  | Perceived enjoyment | Intention to use | n.s. |
|  |  |  |  |  | Perceived ease of use | Intention to use | n.s. |
|  |  |  |  |  | Perceived usefulness | Intention to use | ** |
|  |  |  |  |  | Privacy concern | Intention to use | n.s. |
|  |  |  |  |  | Perceived risk | Intention to use | *** |
|  |  |  |  |  | Perceived trust | Intention to use | ** |
| Oyibo and Morita [75] | Unified theory of acceptance and use of technology | Quantitative | Americas | 242 | Perceived compatibility | Willingness to download app | n.s. |
|  |  |  |  |  | Perceived enjoyment | Willingness to download app | n.s. |
|  |  |  |  |  | Perceived ease of use | Willingness to download app | n.s. |
|  |  |  |  |  | Perceived usefulness | Willingness to download app | n.s. |
|  |  |  |  |  | Privacy concern | Willingness to download app | n.s. |
|  |  |  |  |  | Perceived risk | Willingness to download app | n.s. |
|  |  |  |  |  | Perceived trust | Willingness to download app | n.s. |
| Prakash and Das [27] | Theory of innovation resistance, Theory of distrust | Quantitative | Asia | 194 | Resistance | Intention to use | ***(-) |
| Prakash et al. [76] | Expectation confirmation theory, Trust theory | Quantitative | Asia | 206 | Perceived usefulness | Continuance intention | n.s. |
|  |  |  |  |  | Trust in DCT app | Continuance intention | ** |
|  |  |  |  |  | User satisfaction | Continuance intention | ** |
| Rahimi et al. [77] | Health belief model | Quantitative | Asia | 1031 | Attitude toward technology | Intention to use a contact-tracing app | *** |
|  |  |  |  |  | Fear of COVID-19 | Intention to use a contact-tracing app | *** |
|  |  |  |  |  | Health status | Intention to use a contact-tracing app | n.s. |
| Ross [78] | Regulatory focus theory | Quantitative | Asia | 397 | Prevention focus | Intention to use | * |
|  |  |  |  |  | Privacy concerns | Intention to use | *** |
|  |  |  |  |  | COVID-19 perceived risk | Intention to use | * |
| Saladdin and Handayani [79] | Unified theory of acceptance and use of technology, Health belief model | Quantitative | Asia | 519 | Government involvement | Intentions | ** |
|  |  |  |  |  | Social influence | Intentions | n.s. |
|  |  |  |  |  | Perceived ease of use | Intentions | * |
|  |  |  |  |  | Perceived benefits | Intentions | *** |
|  |  |  |  |  | Facilitating condition | Intentions | *** |
|  |  |  |  |  | Perceived threat | Intentions | *** |
|  |  |  |  |  | Privacy concerns | Intentions | n.s. |
|  |  |  |  |  | System quality | Intentions | *** |
| Scholl and Sassenberg [80] | Social identify theory | Quantitative | Europe | 355 | Identification with government | App acceptance | n.s. |
|  |  |  |  |  | Identification with people around the world | App acceptance | p<0.1 |
|  |  |  |  |  | Identification with social environment | App acceptance | n.s. |
|  |  |  |  |  | Trust | App acceptance | *** |
| Scholz et al. [40] | Protection motivation theory | Quantitative | Europe | 1525 | Actively searching COVID-19 related information in media | Intention to use | n.s. |
|  |  |  |  |  | Social norm for DCT | Intention to use | n.s. |
|  |  |  |  |  | Risk to others of contracting COVID-19 | Intention to use | n.s. |
|  |  |  |  |  | Risk to others of developing severe symptoms | Intention to use | n.s. |
|  |  |  |  |  | Risk to self of spreading the virus | Intention to use | n.s. |
|  |  |  |  |  | Risk to self of developing severe symptoms | Intention to use | n.s. |
|  |  |  |  |  | Risk to self of contracting COVID-19 | Intention to use | * |
|  |  |  |  |  | Believing that oneself already had COVID-19 | Intention to use | n.s. |
|  |  |  |  |  | Response efficacy for DCT | Intention to use | *** |
|  |  |  |  |  | Self-efficacy for DCT | Intention to use | *** |
|  |  |  |  |  | Trust in government | Intention to use | * |
|  |  |  |  |  | Trust in health care system | Intention to use | n.s. |
| Shahidi et al. [81] | Technology acceptance model | Quantitative | Europe | 366 | Goal desire | Intention to use DCT | n.s. |
|  |  |  |  |  | Subjective norms | Intention to use DCT | n.s. |
|  |  |  |  |  | Perceived usefulness | Intention to use DCT | *** |
|  |  |  |  |  | Privacy concerns | Intention to use DCT | *(-) |
| Sharma et al. [82] | Privacy calculus theory, Procedural fairness theory, Hofstede's cultural dimension theory, Theory of planned behavior, Risk calculus theory | Quantitative | Oceania | 714 | Attitude | Adoption intention | *** |
|  |  |  |  |  | Subjective norm | Adoption intention | *** |
|  |  |  |  |  | Privacy self-efficacy | Adoption intention | ** |
| Suh and Li [83] | Cognitive appraisal theory | Qualitative+Quantitative | Asia | 506 | Perceived threat | Adoption intention | ***(-) |
|  |  |  |  |  | Achievement | Continuance intention | n.s. |
|  |  |  |  |  | Challenge | Continuance intention | *** |
|  |  |  |  |  | Deterrence | Continuance intention | n.s. |
|  |  |  |  |  | Loss | Continuance intention | ***(-) |
|  |  |  |  |  | Perceived opportunity | Continuance intention | *** |
| Thenoz et al. [84] | Privacy calculus theory | Quantitative | Europe | 779 | Distrust towards government | Download and activation of DCT | * |
|  |  |  |  |  | Perceived value of using DCT | Download and activation of DCT | * |
|  |  |  |  |  | Personal innovation | Download and activation of DCT | * |
| Tomczyk et al. [85] | Unified theory of acceptance and use of technology, Unified theory of acceptance and use of technology 2, Health belief model, Protection motivation theory, Theory of planned behavior | Quantitative | Europe | 349 | Attitudes | Adoption intention | n.s. |
|  |  |  |  |  | Perceived barriers | Adoption intention | n.s. |
|  |  |  |  |  | Experience | Adoption intention | n.s. |
|  |  |  |  |  | Habit | Adoption intention | n.s. |
|  |  |  |  |  | Hedonic motivation | Adoption intention | *** |
|  |  |  |  |  | Personal norms | Adoption intention | *** |
|  |  |  |  |  | Injunctive social norms | Adoption intention | *** |
|  |  |  |  |  | Descriptive social norms | Adoption intention | n.s. |
|  |  |  |  |  | Perceived ease of use | Adoption intention | n.s. |
|  |  |  |  |  | Perceived usefulness | Adoption intention | n.s. |
|  |  |  |  |  | Price value | Adoption intention | * |
|  |  |  |  |  | Self-efficacy | Adoption intention | n.s. |
|  |  |  |  |  | Perceived controllability | Adoption intention | n.s. |
| Tran and Nguyen [86] | Privacy calculus theory, Risk-risk tradeoff | Quantitative | Americas | 285 | Perceived health risk | App usage | *** |
|  |  |  |  |  | Perceived value | App usage | *** |
|  |  |  |  |  | Perceived privacy risk | App usage | ** |
| Trang et al. [9] | Privacy theory | Quantitative | Europe | 518 | Coronavirus anxiety | Installation intention | ** |
|  |  |  |  |  | Convenience design | Installation intention | ** |
|  |  |  |  |  | Self-benefit appeal | Installation intention | **(-) |
|  |  |  |  |  | Societal-benefit appeal | Installation intention | **(-) |
|  |  |  |  |  | General privacy concern | Installation intention | **(-) |
|  |  |  |  |  | Privacy design | Installation intention | * |
|  |  |  |  |  | IT self-efficacy | Installation intention | ** |
| Trkman et al. [87] | Crisis decision theory | Quantitative | Europe | 401 | Perceived crises severity | Intention to use | *** |
|  |  |  |  |  | Personal benefits | Intention to use | *** |
|  |  |  |  |  | Societal benefits | Intention to use | *** |
|  |  |  |  |  | Societal benefits | Intention to use | *** |
| Velicia-Martin et al. [89] | Technology acceptance model | Quantitative | Europe | 482 | Perceived risk COVID-19 | Behavioral intention to use | *** |
|  |  |  |  |  | Attitudes to apps | Behavioral intention to use | *** |
|  |  |  |  |  | Perceived usefulness | Behavioral intention to use | *** |
|  |  |  |  |  | Privacy concern | Behavioral intention to use | n.s. |
|  |  |  |  |  | Trust | Behavioral intention to use | *** |
| Verpaalen et al. [90] | Psychological reactance theory | Quantitative | Europe | 1480 | Anxiety | DCT use | * |
|  |  |  |  |  | Descriptive norms | DCT use | * |
|  |  |  |  |  | Injunctive norms | DCT use | * |
|  |  |  |  |  | Reactance | DCT use | * |
| Walrave et al. [91] | Health belief model | Quantitative | Europe | 1500 | Perceived susceptibility | Behavioral intention to adopt | n.s. |
|  |  |  |  |  | Perceived severity | Behavioral intention to adopt | n.s. |
|  |  |  |  |  | Perceived benefits | Behavioral intention to adopt | ** |
|  |  |  |  |  | Perceived barriers | Behavioral intention to adopt | **(-) |
|  |  |  |  |  | Self-efficacy | Behavioral intention to adopt | ** |
|  |  |  |  |  | Cues to action | Behavioral intention to adopt | ** |
| Walrave et al. [92] | Protection motivation theory | Quantitative | Europe | 1500 | Performance expectancy | Behavioral intention | * |
|  |  |  |  |  | Effort expectancy | Behavioral intention | n.s. |
|  |  |  |  |  | Social influence | Behavioral intention | * |
|  |  |  |  |  | Facilitating condition | Behavioral intention | * |
|  |  |  |  |  | Innovativeness | Behavioral intention | * |
|  |  |  |  |  | App-related privacy concern | Behavioral intention | *(-) |
|  |  |  |  |  | COVID-19 related stress | Behavioral intention | n.s. |
| Wnuk et al. [41] | Compensatory control model | Quantitative | Europe | 1033 | Lack of control | Attitudes towards radical measures to counteract the pandemic | ** |
|  |  |  |  |  | Personal threat | Attitudes towards radical measures to counteract the pandemic | ** |
|  |  |  |  | 1404 | Endorsement of liberty | Attitudes towards radical measures to counteract the pandemic | **(-) |
|  |  |  |  |  | Lack of control | Attitudes towards radical measures to counteract the pandemic | p=0.5 |
|  |  |  |  |  | Political view-moral | Attitudes towards radical measures to counteract the pandemic | ** |
|  |  |  |  |  | Personal threat | Attitudes towards radical measures to counteract the pandemic | ** |
|  |  |  |  |  | Political view-economic | Attitudes towards radical measures to counteract the pandemic | n.s. |
|  |  |  |  |  | Right-wing authoritarianism | Attitudes towards radical measures to counteract the pandemic | ** |
| Yuduang et al. [93] | Unified theory of acceptance and use of technology 2, Protection motivation theory | Quantitative | Asia | 907 | Facilitating condition | Intention to use | * |
|  |  |  |  |  | Habit | Intention to use | ** |
|  |  |  |  |  | Understanding COVID-19 | Intention to use | * |
|  |  |  |  |  | Hedonic motivation | Intention to use | ** |
|  |  |  |  |  | Social influence | Intention to use | ** |
|  |  |  |  |  | Effort expectancy | Intention to use | * |
|  |  |  |  |  | Performance expectancy | Intention to use | * |
|  |  |  |  |  | Privacy | Intention to use | ** |
|  |  |  |  |  | Self-efficacy | Intention to use | * |
|  |  |  |  |  | Trust | Intention to use | ** |
| Zhang and Vaghefi [42] | Health belief model | Quantitative | Americas | 363 | Perceived benefits of DCT | Continued use intention | *** |
|  |  |  |  |  | Perceived barriers of DCT | Continued use intention | ***(-) |
|  |  |  |  |  | COVID-19 threat severity | Continued use intention | * |
|  |  |  |  |  | COVID-19 threat susceptibility | Continued use intention | * |
|  |  |  |  |  | Self-efficacy to use DCT | Continued use intention | * |
|  |  |  |  |  | Cues to action | Continued use intention | *** |
| van der Waal et al. [88] | Unified theory of acceptance and use of technology, Health belief model | Quantitative | Europe | 1865 | Corona infection others | DCT adoption | n.s. |
|  |  |  |  |  | Perceived barriers | DCT adoption | *** |
|  |  |  |  |  | Monitoring beliefs | DCT adoption | *** |
|  |  |  |  |  | Conspiracy beliefs | DCT adoption | * |
|  |  |  |  |  | Facilitating condition | DCT adoption | *** |
|  |  |  |  |  | Fear beliefs | DCT adoption | * |
|  |  |  |  |  | Social influence | DCT adoption | *** |
|  |  |  |  |  | Effort expectancy | DCT adoption | *** |
|  |  |  |  |  | Performance expectancy | DCT adoption | *** |
|  |  |  |  |  | Perceived benefits | DCT adoption | *** |
|  |  |  |  |  | Technology performance | DCT adoption | n.s. |
|  |  |  |  |  | Societal beliefs | DCT adoption | n.s. |
|  |  |  |  |  | Data safety | DCT adoption | ** |
|  |  |  |  |  | Self-efficacy | DCT adoption | * |
|  |  |  |  |  | Perceived susceptibility | DCT adoption | n.s. |
|  |  |  |  |  | Perceived severity | DCT adoption | n.s. |
|  |  |  |  |  | Perceived susceptibility (others) | DCT adoption | n.s. |
|  |  |  |  |  | Perceived severity (others) | DCT adoption | *** |

*Notes*: DCT means digital contact tracing, * means *p* < 0.05, ** means *p* < 0.01, *** means *p* < 0.001, n.s. means not significant, and (-) means negative direction.

# Supplementary file C. Studies included in meta-analysis

| Studies | Country | Individualism/ Collectivism | Uncertainty/ Avoidance | n | Theory |
| --- | --- | --- | --- | --- | --- |
| Ahmad et al. [43] | China | Low | Low | 320 | Theory of planned behavior, Theory of reasoned action |
| Alkhalifah and Bukar [29] | Nigeria | Low | Low | 309 | Task-technology fit, Technology acceptance model, Protection motivation theory |
| Alshami et al. [30] | Malaysia | Low | Low | 150 | Unified theory of acceptance and use of technology |
| Chan et al. [33] | Malaysia | Low | Low | 401 | Unified theory of acceptance and use of technology 2 |
| Chopdar [46] | India | Low | Low | 307 | Unified theory of acceptance and use of technology, Health belief model |
| Cocosila et al. [48] | Canada | High | Low | 309 | Theory of consumption values |
| Dzandu [50] | United Kingdom | High | Low | 2512 | Technology acceptance model |
| Fox et al. [13] | Ireland | High | Low | 405 | Privacy calculus theory, Social exchange theory |
| Fox et al. [53] | Brazil | Low | High | 1175 | APCO model, Privacy calculus theory, Social contract theory |
| Goyal et al. [15] | India | Low | Low | 850 | Theory of consumption values, Privacy theory |
| Hauff and Nilsson [59] | Sweden | High | Low | 1007 | Privacy calculus theory |
| Jansen-Kosterink et al. [37] | Netherlands | High | Low | 238 | Health belief model, Health belief model |
| Jörling et al. [38] | Germany | High | High | 957 | Technology acceptance model 2 |
| Kaspar [62] | Germany | High | High | 406 | Protection motivation theory |
| Krüger et al. [64] | Germany | High | High | 2682 | Technology acceptance model |
| Lin et al. [68] | Australia | High | Low | 209 | Diffusion innovation theory, Internet users' information privacy concern |
| Meier et al. [17] | Germany | High | High | 952 | Privacy calculus theory |
| Oldeweme et al. [73] | Germany | High | High | 1003 | Uncertainty reduction theory |
| Rahimi et al. [77] | Iran | Low | Low | 1031 | Health belief model |
| Scholl and Sassenberg [80] | Germany | High | High | 355 | Social identify theory |
| Sharma et al. [82] | Fiji | Low | Low | 714 | Privacy calculus theory, Procedural fairness theory, Hofstede's cultural dimension theory, Theory of planned behavior, Risk calculus theory |
| Tomczyk et al. [85] | Germany | High | High | 349 | Unified theory of acceptance and use of technology, Unified theory of acceptance and use of technology 2, Health belief model, Protection motivation theory, Theory of planned behavior |
| Trang et al. [9] | Germany | High | High | 518 | Privacy theory |
| Velicia-Martin et al. [89] | Spain | Low | High | 482 | Technology acceptance model |
| Walrave et al. [91] | Belgium | High | High | 1500 | Health belief model |

# Supplementary file D. Forest plots


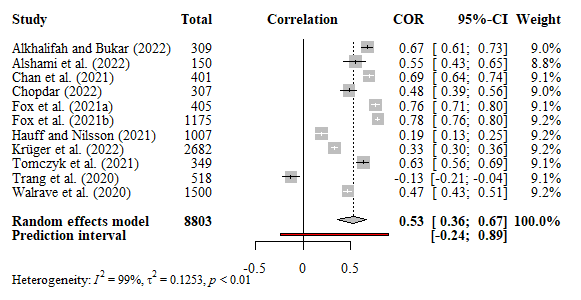


Fig. D1. Forest plot of perceived personal benefits and intention to use


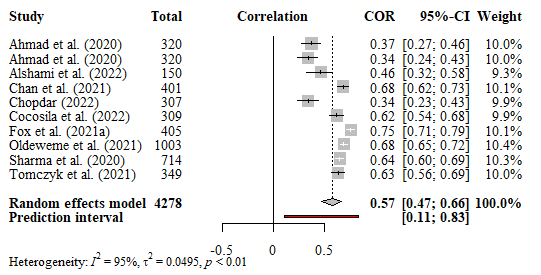


Fig. D2. Forest plot of norms and intention to use


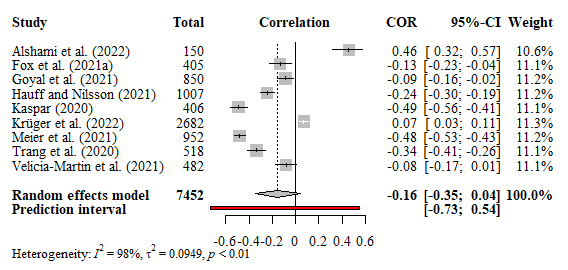


Fig. D3. Forest plot of privacy concerns and intention to use


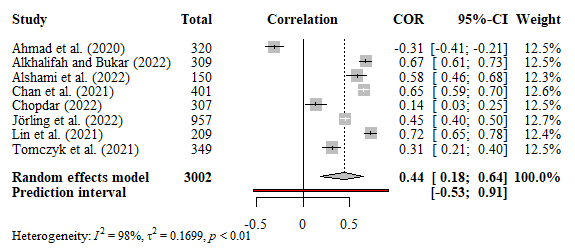


Fig. D4. Forest plot of perceived ease of use and intention to use


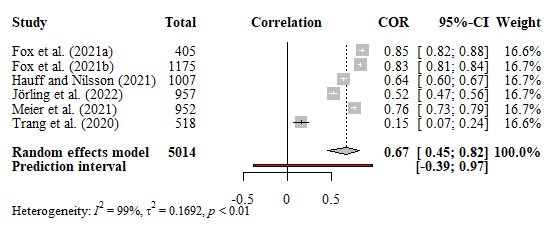


Fig. D5. Forest plot of perceived social benefits and intention to use


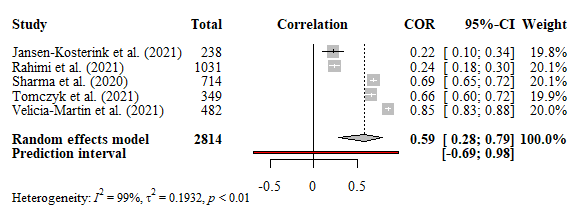


Fig. D6. Forest plot of attitude towards DCT and intention to use


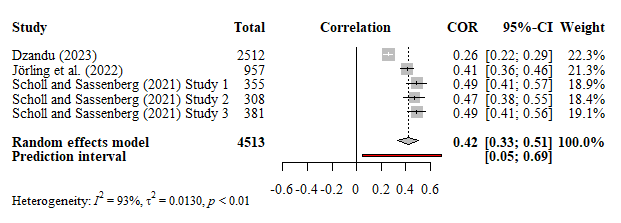


Fig. D7. Forest plot of trust in the government and intention to use


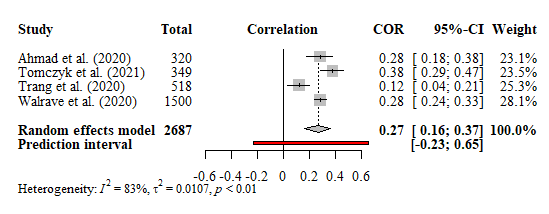


Fig. D8. Forest plot of self-efficacy and intention to use


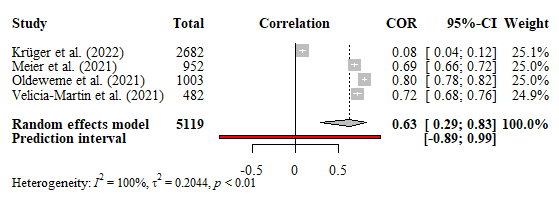


Fig. D9. Forest plot of trust in DCT and intention to use


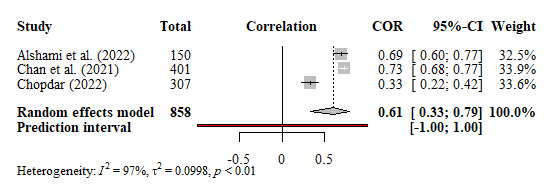


Fig. D10. Forest plot of facilitating conditions and intention to use


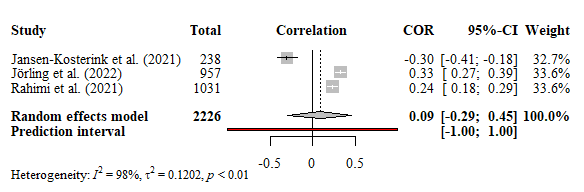


Fig. D11. Forest plot of fear of COVID-19 and intention to use


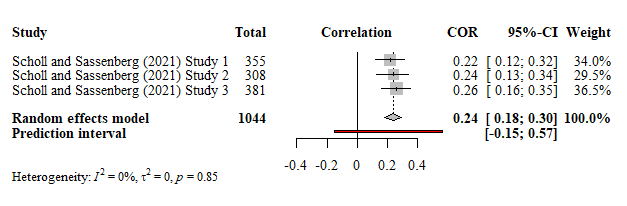


Fig. D12. Forest plot of identification with the members of the government and intention to use


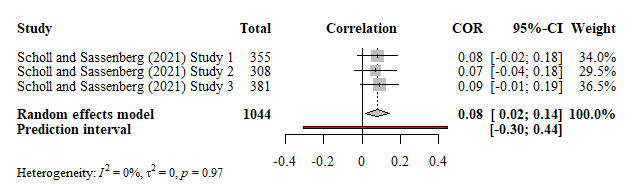


Fig. D13. Forest plot of identification with social environment and intention to use


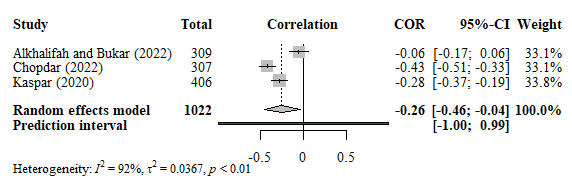


Fig. D14. Forest plot of privacy risks and intention to use
